# CO_2_ Adsorption by Carbon Quantum Dots/Metal Ferrites (M = Co^2+^, Ni^2+^, and Zn^2+^): Electrochemical and Theoretical Studies

**DOI:** 10.1021/acsomega.4c10723

**Published:** 2025-04-03

**Authors:** Alan-Javier Santiago-Cuevas, Cristian-Brayan Palacios-Cabrera, Eduardo Daniel Tecuapa-Flores, Ivan J. Bazany-Rodríguez, Jayanthi Narayanan, Itzia Irene Padilla-Martínez, Carlos Alberto Aguilar, Pandiyan Thangarasu

**Affiliations:** † Facultad de Química, Ciudad Universitaria, Universidad Nacional Autónoma de México (UNAM), 04510 Mexico City, Mexico; ‡ División de Ingeniería en Nanotecnología, Universidad Politécnica del Valle de México, Av. Mexiquense s/n esquina Av. Universidad Politécnica, Tultitlán, Estado de México CP 54910, Mexico; § Instituto Politécnico Nacional Unidad Profesional Interdisciplinaria de Biotecnología (UPIBI-IPN), Ciudad de México, Mexico City C.P 07738, Mexico; ∥ School of Engineering and Sciences, Tecnológico de Monterrey, Atlixcáyotl 5718, Puebla CP 72453, Mexico

## Abstract

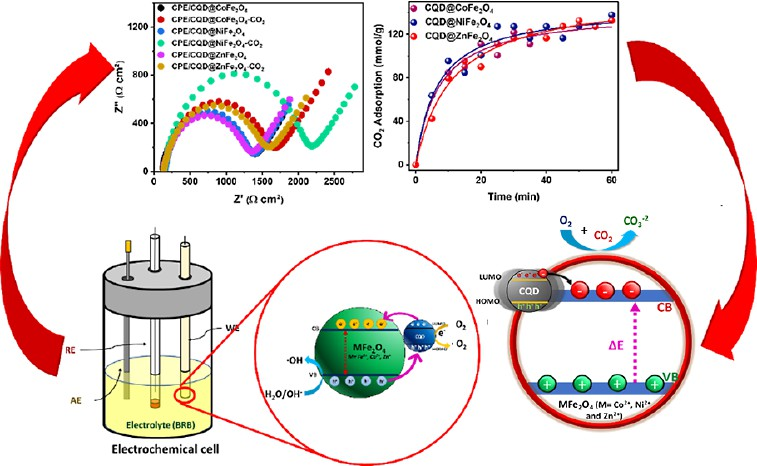

In this study, we investigated the adsorption of CO_2_ by carbon quantum dot-based ferrites (MFe_2_O_4_, M = Co^2+^, Ni^2+^, and Zn^2+^) in the context of industrial CO_2_ emissions and global warming. The ferrites have been characterized using various analytical techniques [X-ray powder diffraction, FTIR, thermogravimetric analysis (TGA), scanning electron microscopy (SEM), transmission electron microscopy (TEM), and X-ray photoelectron spectroscopy (XPS)], showing cubic spinel for CoFe_2_O_4_, reverse cubic spinel for NiFe_2_O_4_, and typical spinel for ZnFe_2_O_4_. A TGA study revealed a significant weight loss around 740–780 °C, indicating structural change occurred with increasing temperature. SEM and TEM images displayed spherical particles with sizes ranging from 10 to 50 nm. XPS confirmed the presence of C, O, and Fe atoms with specific cations (Co^2+^, Ni^2+^, and Zn^2+^). Electrochemical impedance Nyquist diagrams suggest a linear relationship between Z″ (ohm) and Z′ (ohm) at low frequencies, but the semicircular loop obtained was significantly increased at higher frequencies. This suggests that the charge transfer resistance (*R*_CT_) at the electrode boundaries (interface) is much higher than at low frequencies, indicating the resistance per area was 1853 Ω cm^2^ for carbon paste electrodes (CPE)/CoFe_2_O_4_ and it decreased to 1652 Ω cm^2^ for CPE/NiFe_2_O_4_ and 1672 Ω cm^2^ for CPE/ZnFe_2_O_4_. However, improved electron transfer with lower resistance was seen due to the composite nature of the samples (CQDs@MFe_2_O_4_), revealing a lower resistance (1163 Ω cm^2^) for CQD@MFe_2_O_4_–CO_2_ as compared to 1567 Ω cm^2^ for MFe_2_O_4_. Thus, the adsorption of CO_2_ was studied electrochemically, and interaction between ferrates with CO_2_ was enhanced by the presence of CQDs in the samples. This is consistent with the adsorption of CO_2_ with the samples as it follows the Langmuir pseudo-second-order kinetics (*k* = 4.9, *qe* = 121.93 for CQD@CoFe_2_O_4_, *k* = 2.9, *qe* = 156.52 for CQD@NiFe_2_O_4_, and *k* = 3.0, *qe* = 141.71 for CQD@ZnFe_2_O_4_), and the data show that the adsorption efficiency has been decreased by around 1.0% after 7–8 cycles. Lastly, density functional theory analysis demonstrated the interaction of CO_2_ on the surface of the ferrites, deforming the CO_2_ linearity, which leads to a subsequent C–O interaction to form carbonate.

## 1 Introduction

Carbon dioxide emissions associated with global warming significantly threaten the environment and sustainable development.^1,2^ Controlling CO_2_ emissions from correlative industries is essential in mitigating global warming,^3^ since CO_2_ contributes around 77% of greenhouse gases (CO_2_ atmospheric lifetime is approximately 300 years).^4^ Carbon capture and storage has been considered at different stages in industrial processes to reduce CO_2_ emissions,^5,6^ even though other methods, such as physical/chemical sorption,^7,8^ membrane-based separation,^9^ solar thermochemical splitting, photocatalytic conversion of CO_2,_^10,11^ and amine solvent-based CO_2_ absorption^12^ are being considered. So, the adsorption of CO_2_ through promising sorbent is essential, for which the following metal oxides, such as Li_4_SiO_4_,^13−16^ Li_5_FeO_4,_^17^ Li_5_AlO_4_,^18^ Li_2_CuO_2,_^19^ Li_2_ZrO_3,_^20^ LiFeO_2,_^21−23^ Li_2_TiO_3,_^24^ Na_2_SiO_3,_^25^ and NaFeO_2_^26^ were explored. Among the samples, Li_5_FeO_4_ has efficiently improved the CO_2_ capture at a low temperature (150–400 °C).^27^ Furthermore, Ba_3_Fe_2_O_6_ and Ba_5_Fe_2_O_8_ are also tested for carbon dioxide capture, showing that chemical looping oxygen uncoupling supports a high initial CO_2._^28^

A chemical looping study associated with thermochemical splitting, where the metal oxides are actively involved in dissociating CO_2_ into CO, is an attractive and alternative method due to its low cost and high efficiency.^29−31^ In particular, the reduction of metal oxides plays a role in forming oxygen vacancies; oxygen-vacant materials also regain the lost oxygen during oxidation.^10,32−35^ Thus, Co, Cu, Co, and Mn codoped spinel ferrites have been considered effective chemical looping for CO_2_ thermal splitting because of their oxygen carrier capacity.^10,36^ In the metal oxide looping combustion, metal oxide provides oxygen to the combustion, to which Ca_2_Fe_2_O_5_, CuFe_2_O_4_, FeCuAlO_4_, NiFe_2_O_4_, and CuMn_2_O_4_ and NiFe_2–*x*_Al_*x*_O_4_ (*x* = 1.0) has been tested to remove carbon (CO + CO_2_).^37−41^ In addition, the spinel-ferrites such as Cu_*x*_Mn_1–*x*_Fe_2_O_4_ (*x* = 0.3, 0.5, 0.6, and 0.7), Cu_*x*_Mg_1–*x*_Fe_2_O_4_ and NiFe_2_O_4_ have been effectively used as oxygen carrier,^42−44^ exhibiting better performance than individual Fe_2_O_3_. Iron oxides (maghemite Fe_2_O_3_ and magnetite Fe_3_O_4_) are attractive because they are superparamagnetic, catalytic, and nontoxic,^45−48^ and also other oxides (Fe_2_O_3_, Mn_3_O_4_, and CuO) are also considered oxygen carriers.^49−51^ Nonetheless, for Fe_3_O_4_ NPs, the surface spin imbalance in an internal structure dramatically influences the magnetic properties (spinel-type ferrite); the stability and size of these particles get aggregated easily in the suspension medium because of their high magnetic and surface energy.^52^ The doping of metals on magnetite MFe_2_O_4_ (M = Mn, Co, Ni, Zn, Mg, etc.) has increased their stability and magnetic and catalytic properties. For spinel ferrites (MFe_2_O_4_; M = Mg, Zn, Co, Ni, Mn, etc.), there are two cation sites {(M^2+^_1–*c*_Fe_*c*_^3+^)_A_[M_*c*_^2+^ Fe_2–*c*_^3+^]_B_O_4_, where *c* = inversion parameter, giving a fractional number which signifies the occupational disorder of divalent cations in octahedral sites.^53−56^

The *h*^+^ carriers create the crystal vacancy defects in the oxide structure,^57^ and to keep the charge neutrality, the substitution of cations is employed, as seen for LaFeO_3_, where the replacement of La^3+^ or Fe^3+^ by divalent cations (Pb^2+^, Mg^2+^, Ba^2+^, Co^2+^, Sr^2+,^ and Ca^2+^) has occurred to increase the *h*^+^ concentration in the valence band (VB),^58,59^ showing that the oxygen vacancies created by modified metal oxides play a crucial role in CO_2_ detection and have enhanced the degree of adsorption of CO_2_. The reversible reaction of oxygen vacancies within the lattice of metal oxide is associated with oxygen species (O^–^ or O^2–^), which withdraw electrons from the conduction band (CB) and involve the reduction of CO_2_. The replacement of Fe^3+^ ions by divalent metal could generate crystal defects, and it can randomly distribute the oxygen vacancies that support the adsorption of CO_2_.^60^ So, the creation of abundant oxygen vacancies in ferrates is challenging work. In the previous works, several materials have been explored for the adsorption of CO_2,_ and it reported that ZF/rGO-MEA30 (zinc ferrite/amine-functionalized reduced graphene oxide nanocomposites)^61^ BaNi_2_Fe_16_O_27_,^62^ Bi_0.5_Sr_0.5_Fe_1–*x*_Ta_*x*_O_3-δ_ (tantalum doped bismuth–strontium ferrite),^63^ CF/rGO (Copper ferrite/reduced graphene oxide)^64^ for the treatment of CO_2_. Li_5_FeO_4_, Ba_3_Fe_2_O_6_, and Ba_5_Fe_2_O_8_ have been tested for CO_2_ capture.^28,65^ Furthermore, KOH/carbonized pollen, NbOFFIVE-1-Ni, and BaNi_2_Fe_16_O_27_ adsorbed CO_2_ efficiently.^66,67^ So, the modified ferrites supported by carbon quantum dots (CQD) are expected to increase CO_2_ adsorption. Furthermore, the adsorption of CO_2_ by different ferrates such as CQD@MFe_2_O_4_ (M = Co^2+^, Ni^2+^, and Zn^2+^) using electrochemical methods is exciting and a novelty in carbon capture; the deposition of CQD on metal oxide-based-material is growing research; thus, CQD-based ferrites (CQD@MF_2_O_4_, M = Co^2+^, Ni^2+^, or Zn^2+^) are prepared and studied for the CO_2_ adsorption properties; furthermore, the interaction of CO_2_ with the ferrates by density functional theory (DFT) is interesting. To the best of our knowledge, the structural optimization, molecular orbital (MO), and density of states (DOS) of different ferrites are not found in the literature.

## 2 Results and Discussion

### 2.1 FT-IR Studies

FT-IR spectroscopy was recorded for all samples MFe_2_O_4_ and CQD@MFe_2_O_4_ (Figure S1), and vibrational characteristic peaks corresponding to spinel nano ferrites were observed.^68^ CQD@CoFe_2_O_4_, which displayed two peaks at 1040 and 1090 cm^–1^, potentially associated with −C–O–M vibrations.^69^ FTIR can predict the characteristics of M–O stretching and M–O–Fe (bond-stretching) (M = Co^2+^, Ni^2+^, or Zn^2+^) vibrations; for example, MFe_2_O_4_ and CQD@MFe_2_O_4_ have exhibited a vibrational peak in the 500 to 750 cm^–1^ range,^68^ consisting of the previous studies.^70,71^ The characteristic peak (533 cm^–1^) for zinc ferrites was observed for Fe–O, assigning octahedral M–O bonds.^70^ while the signal 477 cm^–1^ is attributed to the octahedral Zn^2+^(Zn–O) stretching vibration.^71^ Additionally, small splitting peaks between 1600 and 800 cm^–1^ originating from Zn–O–Fe (bond-stretching) were seen confirming the existence of the tetrahedral building units. For nickel ferrite, two absorption bands (450 and 650 cm^–1^) correspond to the metal–oxygen intrinsic stretching vibrations at the octahedral site, M (octahedral) ↔ O and tetrahedral metal–oxygen stretching, M (tetrahedral) ↔ O, respectively. Only two absorption bands at 420 and 600 cm^–1^ corresponding to the octahedral and tetrahedral metal–oxygen were seen. For CoFe_2_O_4_, the IR peaks were detected at 449 and 535 cm^–1^, corresponding to Fe–O and M–O vibrations, respectively. For NiFe_2_O_4_, the IR signals were observed at 449 and 441 cm^–1^, while for ZnFe_2_O_4_, they appeared at 443 and 522 cm^–1^. For the CQD composites, a slight shift of peaks for the Fe–O and M–O peaks was seen, resulting in 457 and 529 cm^–1^ for CQD@CoFe_2_O_4_; 437 and 548 cm^–1^ for CQD@NiFe_2_O_4_; and 443 and 523 cm^–1^ for CQD@ZnFe_2_O_4_. The shift can be attributed to the interaction between the CQDs and the ferrites.

### 2.2 Raman Spectra

**Figure 1:**
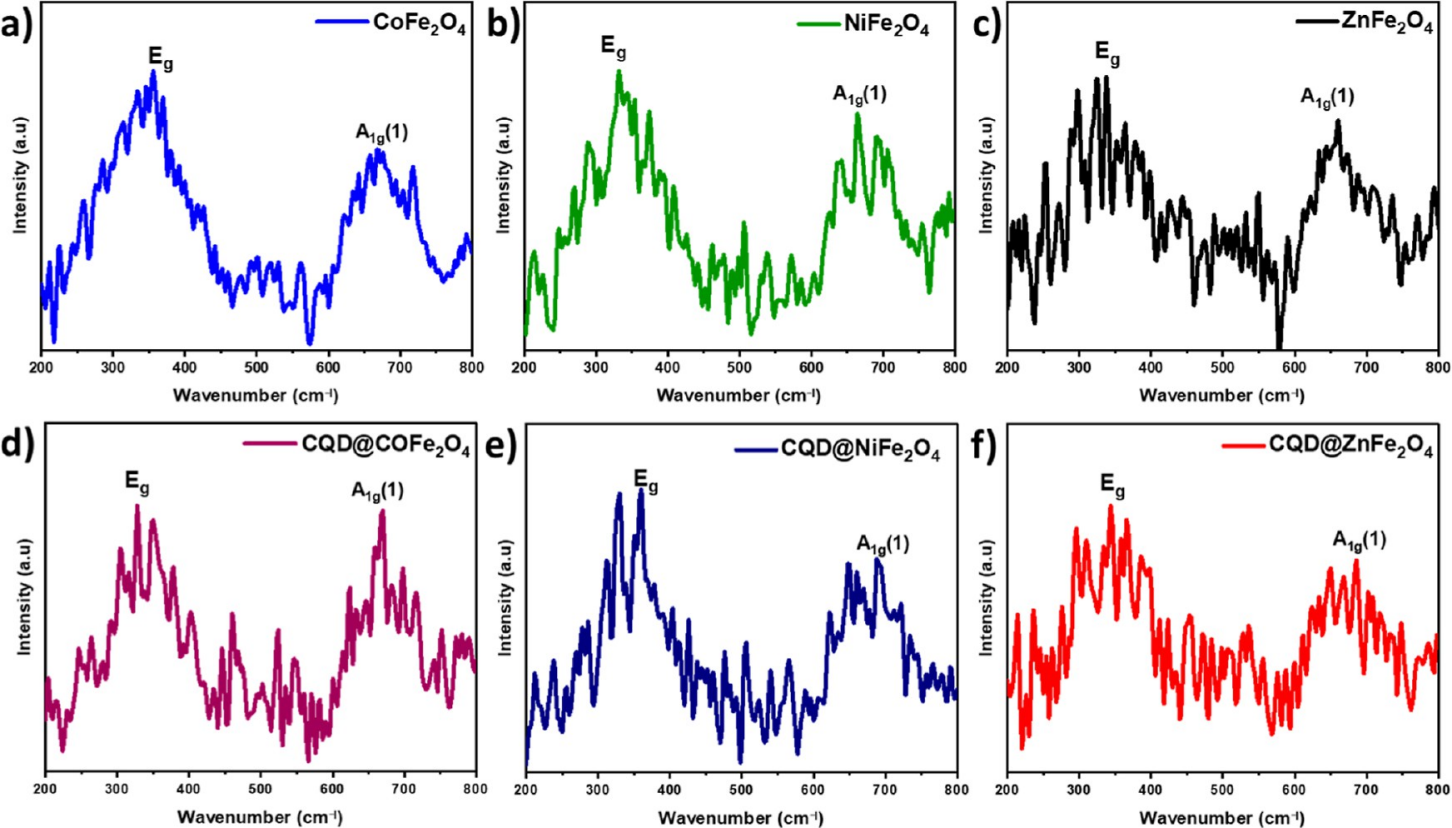
Raman spectra of the samples: (a) CoFe_2_O_4_; (b) NiFe_2_O_4_; (c) ZnFe_2_O_4_; (d) CQD@CoFe_2_O_4_; (e) CQD@NiFe_2_O_4_; and (f) CQD@ZnFe_2_O_4_.

Raman spectra (200–3500 cm^–1^) were recorded for MFe_2_O_4_ and CQD@MFe_2_O_4_, showing the prominent M-O vibration modes (Figure 1);^72^ specifically, the *E*_g_ peak was observed around 350 cm^–1^ for the samples, namely, 355 cm^–1^ for CoFe_2_O_4_, 327 cm^–1^ for CQD@CoFe_2_O_4_, 336 cm^–1^ for NiFe_2_O_4_, 359 cm^–1^ for CQD@NiFe_2_O_4_, 338 cm^–1^ for ZnFe_2_O_4_, and 345 cm^–1^ for CQD@ZnFe_2_O_4_, representing the asymmetrical bending of O-M^3+^ in octahedral B sites.^73^ Other asymmetric vibrations of A_1g_ appeared at around 700 cm^–1^. For example, 668 cm^–1^ for CoFe_2_O_4_, 669 cm^–1^ for CQD@CoFe_2_O_4_, 664 cm^–1^ for NiFe_2_O_4_, 689 cm^–1^ for CQD@NiFe_2_O_4_, 660 cm^–1^ for ZnFe_2_O_4_, and 689 cm^–1^ for CQD@ZnFe_2_O_4_ are observed associated with the O–M^2+^ stretching in tetrahedral arrangements (A sites). For all ferrites, the M–O bonding in the octahedral structure is significant, suggesting a certain degree of inversion, namely, the migration of M^2+^ from tetrahedral to the octahedral site occurred.^74,75^

### 2.3 Thermogravimetric Analysis

**Figure 2:**
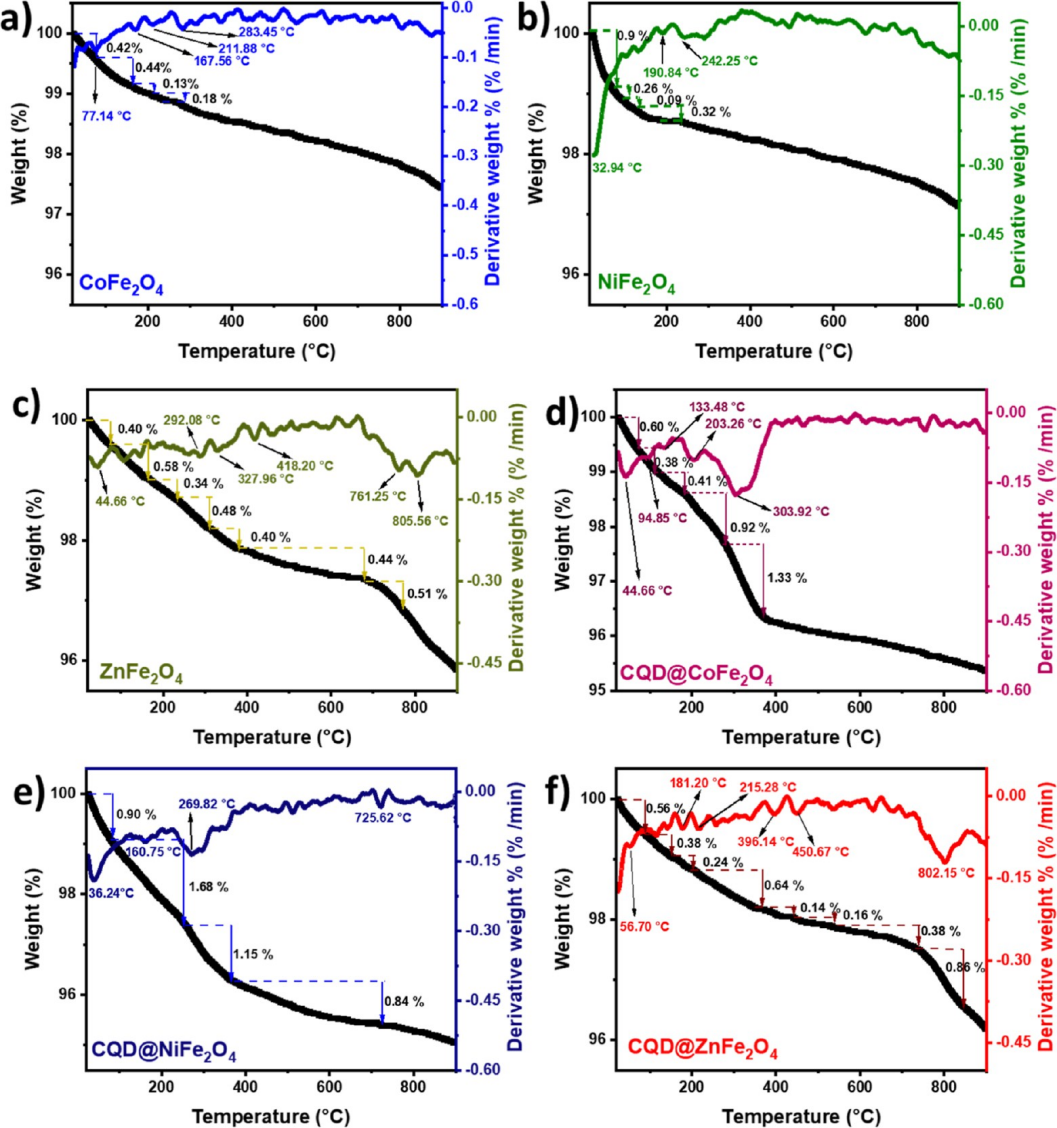
TGA of the samples: (a) CoFe_2_O_4_, (b) NiFe_2_O_4_, (c) ZnFe_2_O_4_, (d) CQD@CoFe_2_O_4_, (e) CQD@NiFe_2_O_4_, and (f) CQD@ZnFe_2_O_4_.

The thermal stability of the samples was analyzed in the range of 180–900 °C, showing the step of weight loss (Figure 2);^76−78^ there is a loss of water molecules (80–156 °C) and then a gradual weight loss of residual functional group with increasing temperature ∼290 °C was seen. A significant weight loss that occurred around 740–780 °C is attributed to the structural change of the ferrites, resulting in the decomposition of CoFe_2_O_4_ (2.56%), NiFe_2_O_4_ (2.85%), and ZnFe_2_O_4_ (3.77%) was observed. For CQD/MFe_2_O_4_, a similar pattern of weight loss (water and the residual components) was noticed; the decomposition of CDQ was also seen around 350–390 °C. The weight loss was 4.64% for CQD@CoFe_2_O_4_, 4.95% for CQD@NiFe_2_O_4_, and 4.10% for CQD@ZnFe_2_O_4_.

### 2.4 X-ray Powder Diffraction (XRD) Analysis

**Figure 3:**
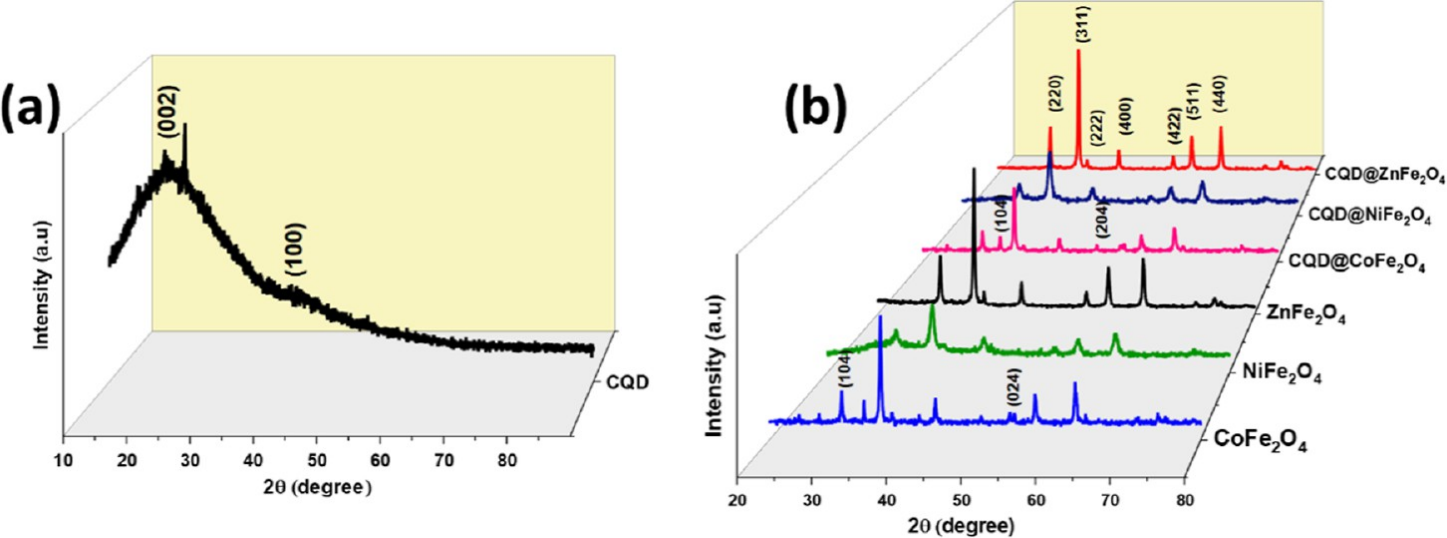
XRD recorded at 2θ: (a) CQDs and (b) MFe_2_O_4_ NPs and CQD@MFe_2_O_4_ (M = Co^2+^, Ni^2+^, and Zn^2+^).

X-ray powder diffraction (XRD) studies performed for CoFe_2_O_4_, NiFe_2_O_4_, ZnFe_2_O_4,_ CQD@CoFe_2_O_4_, CQD@NiFe_2_O_4_, CQD@ZnFe_2_O_4_, and CQDs (Figure 3 and Table S1) show that for CQD, at 2θ (low angle), the characteristic peaks of graphitic carbon were obtained (a broad peak at 19.72° and a weak signal at 41.483°), assigning the (002) and (100) planes, respectively.^79,80^ For CoFe_2_O_4_, the peaks correspond to a monophasic cubic spinel observed (JCPDS card no. 5-667)^81^ as the signals at 35.47, 30.26, 35.47, 43.35, 53.81, 57.37, 63.01, and 74.62° have appeared, corresponding to the crystal planes of (311), (220), (311), (222), (400), (422), (511), (400), and (533), respectively. Two peaks (33.138 and 49.460°) correspond to hematite (JCPDS 33-0664), showing that the sample presents fine crystallinity.^82^ For NiFe_2_O_4_, a reverse cubic spinel structure was obtained as the XRD data are well matched with the standard (JCPDS: 74-2081);^83^ however, the signal at 35.56° (311) has appeared with low intensity, and others 30.2, 35.57, 43.24, 53.69, 57.18, and 62.79° have been assigned to the crystalline planes (220), (222), (400), (422), (511), and (400), respectively. For ZnFe_2_O_4_, the diffraction peaks at 2θ are 29.93, 35.24, 36.85, 42.85, 53.17, 56.67, 62.19, and 73.64° are originated from the planes (220), (311), (222), (400), (422), (511), (440), and (533), respectively.^84^ The XRD planes of CQD in the ferrite composites may not be clearly visible due to their amorphous or semicrystalline nature, producing a very weak diffraction peak caused by their nanometric size associated with significant peak broadening. Additionally, the high intensity of well-defined peaks from the ferrite can overshadow the weak carbon dots (CDs) signals, especially if they are present in low concentrations or if interactions within the composite increase their structural disorder.^85,86^

The size of particles, which was calculated by the Scherrer equation (eq 1) after considering the intense peak of (311), is as follows: 30.05 for CoFe_2_O_4_, 13.43 for NiFe_2_O_4_, 24.97 nm for ZnFe_2_O_4_, 23.30 nm for CQD@CoFe_2_O_4_, 13.39 nm for CQD@NiFe_2_O_4_, and 24.68 nm for CQD@ZnFe_2_O_4_. Generally, the peaks of NiFe_2_O_4_ and CQD@NiFe_2_O_4_ have been shifted to higher angles, followed by CoFe_2_O_4_ and CQD@CoFe_2_O_4_ and then to ZnFe_2_O_4_, CQD@ZnFe_2_O_4_. A comparative analysis of the XRD data with those reported is presented (Table 1), showing that the assignment of the peaks is consistent with the present data.
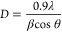
1λ = X-ray wavelength (0.154 nm); β = maximum mean full-width (fwhm); and θ = diffraction angle in radians. In addition, they calculated the nanostructural parameters of MFe_2_O_4_ (Table S2).

**Table 1:** Crystalline Feature of MFe_2_O_4_ (M = Co^2+^, Ni^2+^, and Zn^2+^) with Other Systems

method	M–Fe_2_O_4_ composition	crystal phase	plane	2θ angle (°)	ref
hydrothermal	CoFe_2_O_4_	monophasic cubic spinel	311	35.47	present work
hydrothermal	NiFe_2_O_4_	reverse cubic spinel	311	35.56	present work
hydrothermal	ZnFe_2_O_4_	reverse cubic spinel	311	35.24	present work
hydrothermal	CQD@CoFe_2_O_4_	monophasic cubic spinel	311	35.49	present work
hydrothermal	CQD@NiFe_2_O_4_	reverse cubic spinel	311	35.62	present work
hydrothermal	CQD@ZnFe_2_O_4_	reverse cubic spinel	311	35.25	present work
coprecipitation	Cu–CoFe_2_O_4_–PC	monophasic cubic spinel	311	35.80	(87)
hydrothermal	alginate/MXene/CoFe_2_O_4_	reverse cubic spinel	311	35.41	(88)
hydrothermal	CoFe_2_O_4_–NC/Li_2_S_6_	monophasic cubic spinel	311	35.5	(89)
solvothermal	Ni_3_S_2_/CoFe_2_O_4_	monophasic cubic spinel	311	35.45	(90)
hydrothermal	NiFe_2_O_4_/RGO	reverse cubic spinel	311	35.70	(91)
hydrothermal	NiFe_2_O_4_/g-C_3_N_4_	reverse cubic spinel	311	35.68	(92)
solvothermal	NiFe_2_O_4_	reverse cubic spinel	311	35.70	(93)
microwave	NiFe_2_O_4_	reverse cubic spinel	311	35.99	(94)
hydrothermal	H–ZnFe_2_O_4_. α-Fe_2_O_3_	cubic inverse spinel	311	35.20	(95)
sol–gel	p-CaFe_2_O_4_@n-ZnFe_2_O_4_	cubic inverse spinel	311	35.30	(96)
microwave	ZnFe_2_O_4_	cubic inverse spinel	311	36.65	(94)
chemical bath deposition	Fe_3_O_4_@ZnFe_2_O_4_	cubic inverse spinel	311	35.42	(97)

### 2.5 Scanning Electron Microscopy

Scanning electron microscopy (SEM) images of samples have been observed using secondary electrons and identified the morphology of CoFe_2_O_4_ (×9500), NiFe_2_O_4_ (×16,000), and ZnFe_2_O_4_ (×15,000). The results show spherically round-shaped particles dispersed in a porous nature; however, the particles have been agglomerated due to their paramagnetic properties. Similarly, the round-shaped particles for CQD@CoFe_2_O_4_ (×18,000), CQD@NiFe_2_O (×14,000), and CQD@ZnFe_2_O_4_ (×17,000) are observed, distributing CQDs over the metal oxide surface. The composition of the elements was determined by EDS (Table S3) and compared with theoretical values (Table S4 and Figure S2). The Fe/Co, Fe/Ni, and Fe/Zn and 1.75 ratios were 2.36, 1.93, and 1.75, respectively, coinciding with the theoretical elemental composition.

**Figure 4:**
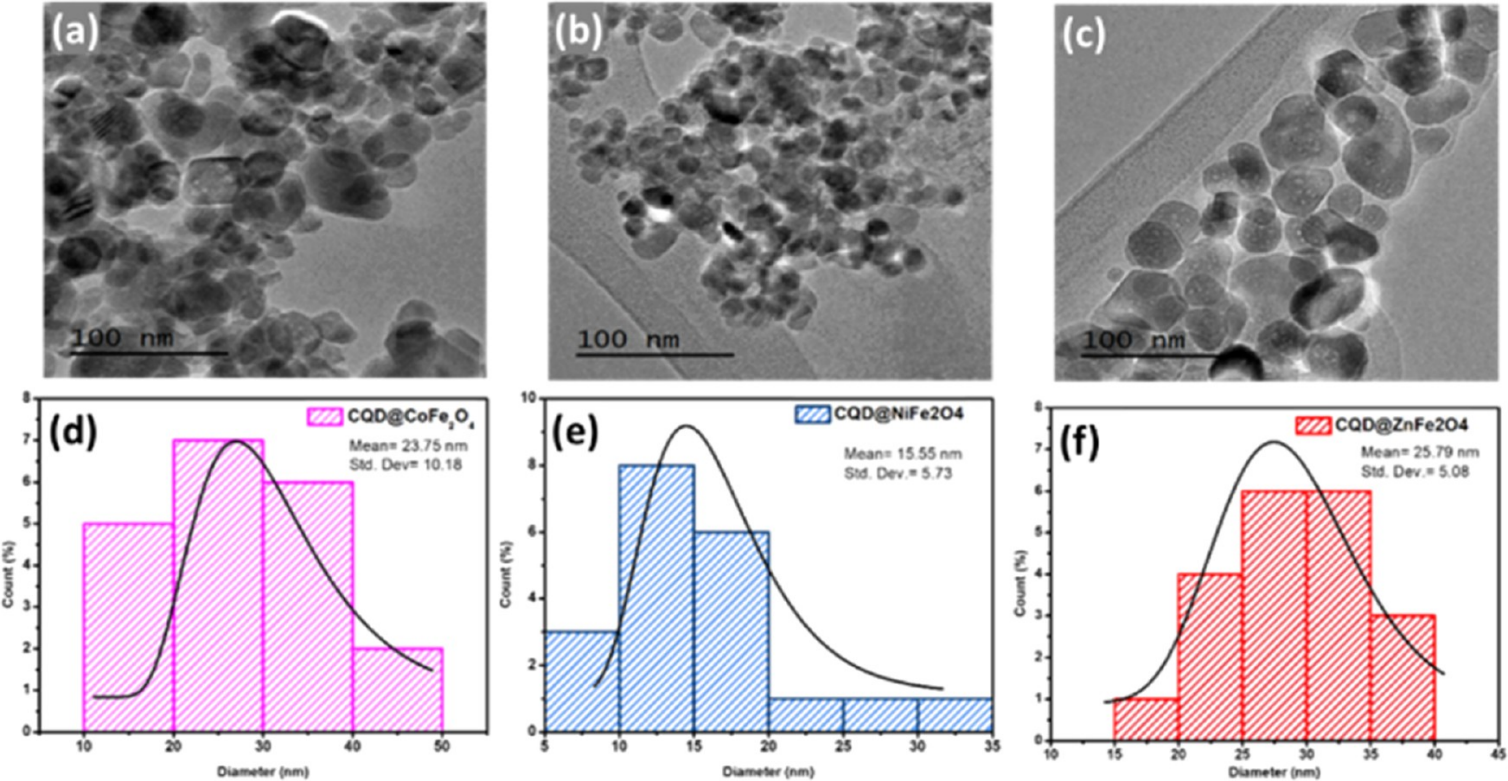
Transmission Electronic Microscopy (TEM images in darkfield): (a) CQD@CoFe_2_O_4_, (b) CQD@NiFe_2_O_4_, (c) CQD@ZnFe_2_O_4_; histogram diameter: (d) CQD@CoFe_2_O_4_, (e) CQD@NiFe_2_O_4_, and (f) CQD@ZnFe_2_O_4._.

EDS analysis reveals the presence of carbon and oxygen signals in the CQD-composite materials and confirms the successful incorporation of CQDs onto the ferrite surfaces (19.70 wt % for CQD@CoFe_2_O_4_, 8.90 wt % for CQD@NiFe_2_O_4_, and 17.90 wt % for CQD@ZnFe_2_O_4_) (Figure S3). It is important to note that the CQDs were not visually detectable in the SEM images due to their size, which is below the resolution limit of conventional SEM. Instead, the presence of CQDs was indirectly inferred from the EDS and changes in surface morphology of the ferrite particles, as seen in Figure 4d–f, where smaller aggregates and smoother surfaces are observed compared to the pristine ferrites.

### 2.6 Transmission Electronic Microscopy

The images of TEM (Figure 4) show the size and morphology of CQD@CoFe_2_O_4_, CQD@NiFe_2_O_4_, and CQD@ZnFe_2_O_4_, observing that the samples have existed in a quasi-circular shape. The size of particles was determined to be ∼10–50 nm for CQD@CoFe_2_O_4_, ∼5–35 nm for CQD@NiFe_2_O_4,_ and ∼15–40 nm for CQD@ZnFe_2_O_4_ (Figure 4a–e). The size and dispersion of particles are associated with their paramagnetic properties, which promote particle agglomeration; as a result, there is an irregular distribution of the CQD (∼3–5 nm) over the CQD@ZnFe_2_O_4_ surface.

**Figure 5:**
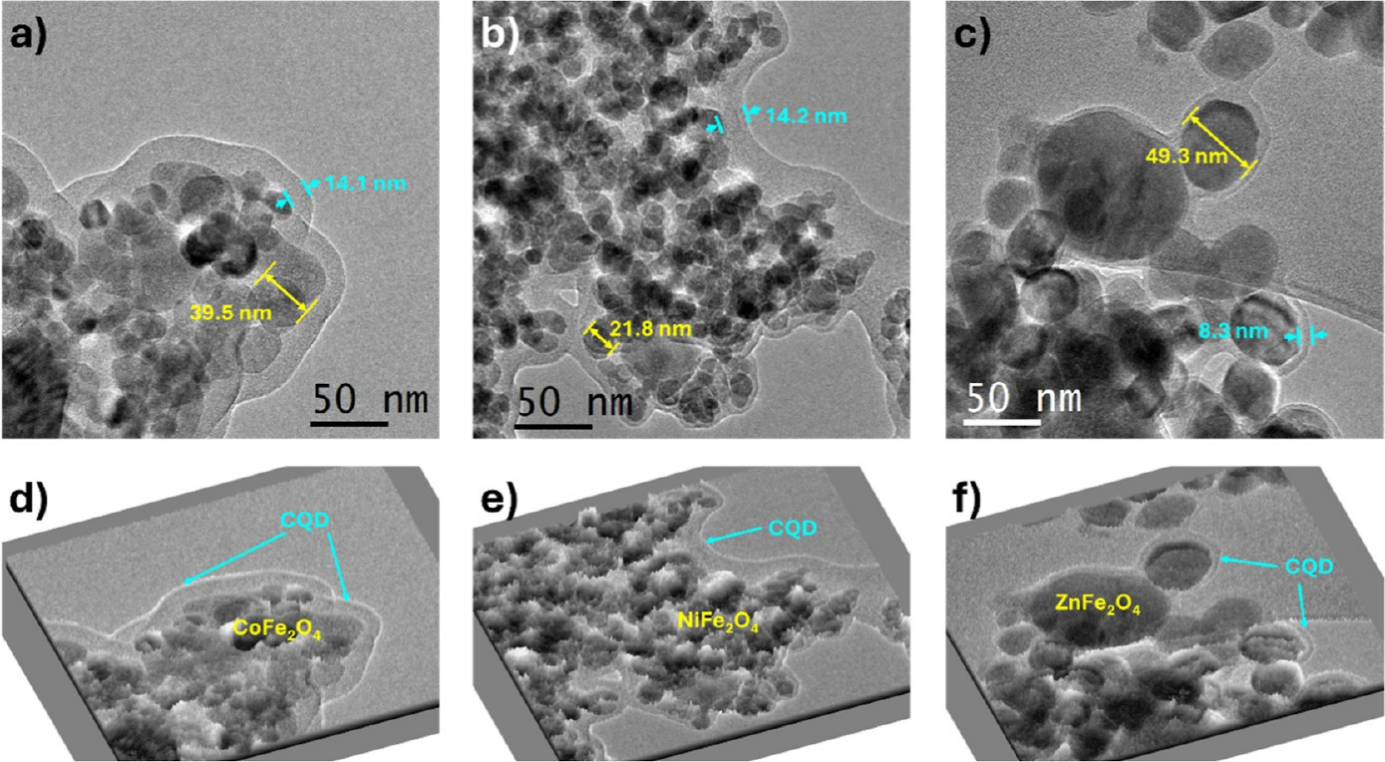
TEM (in brightfield): (a) CQD@CoFe_2_O_4_; (b) CQD@CoFe_2_O_4_; and (c) CQD@CoFe_2_O_4_. Surface TEM mapping for the sample: (d) CQD@CoFe_2_O_4_; (e) CQD@CoFe_2_O_4_; and (f) CQD@CoFe_2_O_4_.

The presence of CQD over the MFe_2_O_4_ surface was confirmed by TEM studies, observing a difference in the morphology. For example, for CQD@CoFe_2_O_4_, in the electronic images, the dense particles of round/irregular morphology correspond to ferrites (size range 30–50 nm) and were surrounded by CQD (Figure 5a); on the ferrite surface, a homogeneous thin layer of CQD with a thickness of around 14 nm was found (Figure 5d). For nickel-based materials (Figure 5b), the metal oxide (size of around 20–30 nm) is covered by a smaller CQD coating, seeing a thickness similar to the one observed for CQD@CoFe_2_O_4_ (Figure 5e). The Zn ferrites are presented in a larger size (above 49 nm) and have round morphology and smooth edges (Figure 5c). However, the CQD coating was not as effective, resulting in the average thickness of the organic coating being less than 10 nm, which was not even noticeable in some zones (Figure 5f).

### 2.7 High-Resolution TEM

**Figure 6:**
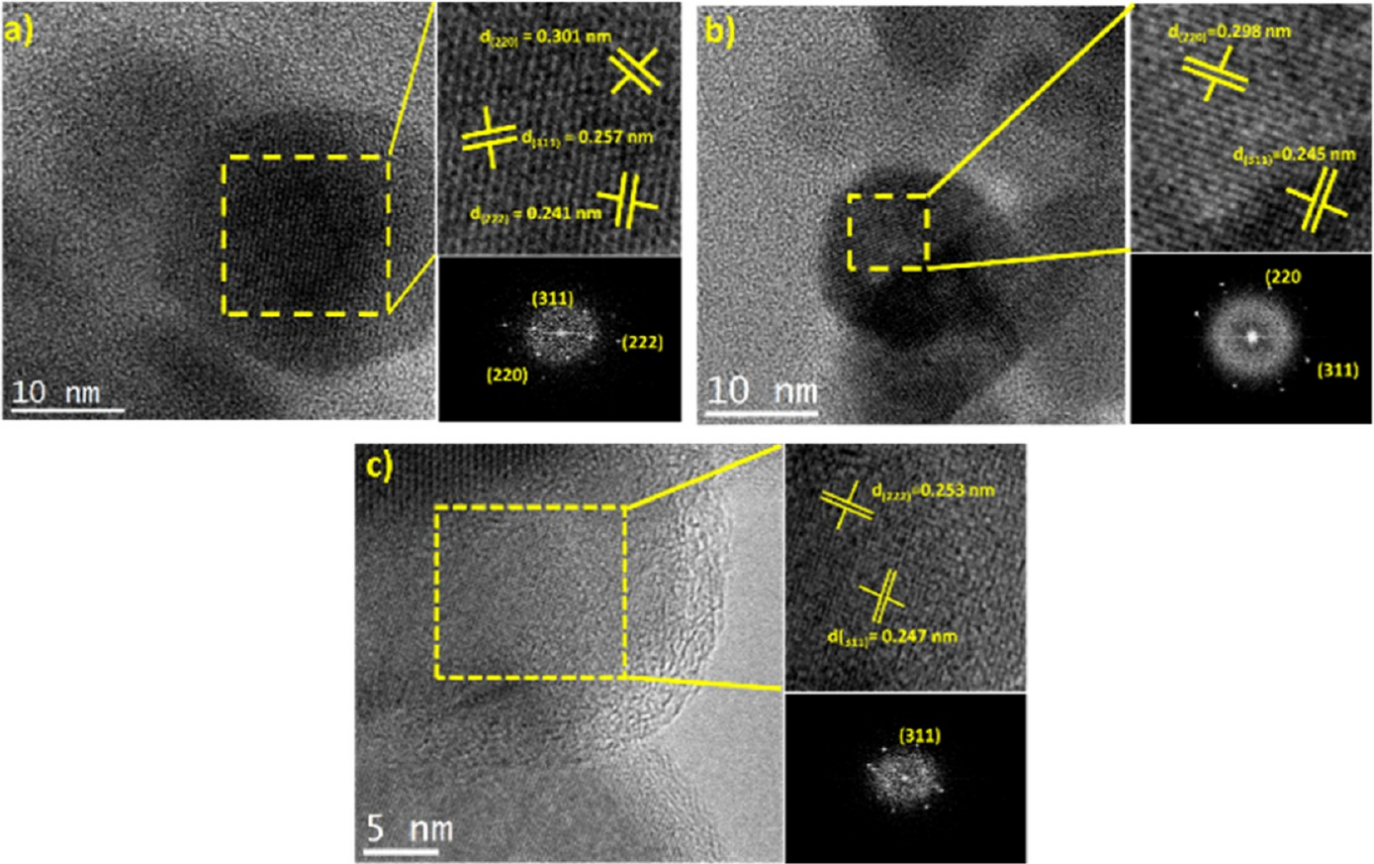
High-resolution TEM images: (a) CQD@CoFe_2_O_4_, (b) CQD@NiFe_2_O_4_, and (c) CQD@ZnFe_2_O_4_.

A homogeneous distribution of the nanoparticles was observed in high-resolution TEM, consisting of TEM analysis. The existence of the crystalline nature in the samples such as CQD@CoFe_2_O_4_, CQD@NiFe_2_O_4_, and CQD@ZnFe_2_O_4_ (Figure 6) was observed, agreeing with the interplanar distance (*d*); for example, for CQD@CoFe_2_O_4_, the *d*(220) = 0.301 nm, *d*(311) = 0.257 nm, *d*(222) = 0.241 nm has resulted. For CQD@NiFe_2_O_4_, the resulting *d* (220) = 0.298 nm, and *d*(311) = 0.245 nm; for CQD@ZnFe_2_O_4_, *d*(222) = 0.253 nm, *d*(311) = 0.247 nm was seen. A slight change in the interplanar distance was caused by the size and type of cations (Co^2+^, Ni^2+^, and Zn^2+^) deposited in the ferrites since the radius of Co^2+^ (0.745 Å) is considerably larger than that of Ni^2+^ (0.69 Å) and Zn^2+^(0.60 Å). Thus, a loss of oxygen ions in the sublattice of Ni^2+^ and Zn^2+^ was caused due to a decrease in the lattice constant.^98−100^ The results are consistent with the formation of well-crystallized spinels, observing an orderly structural arrangement of atoms.^101,102^

### 2.8 X-ray Photoelectron Spectroscopy

**Figure 7:**
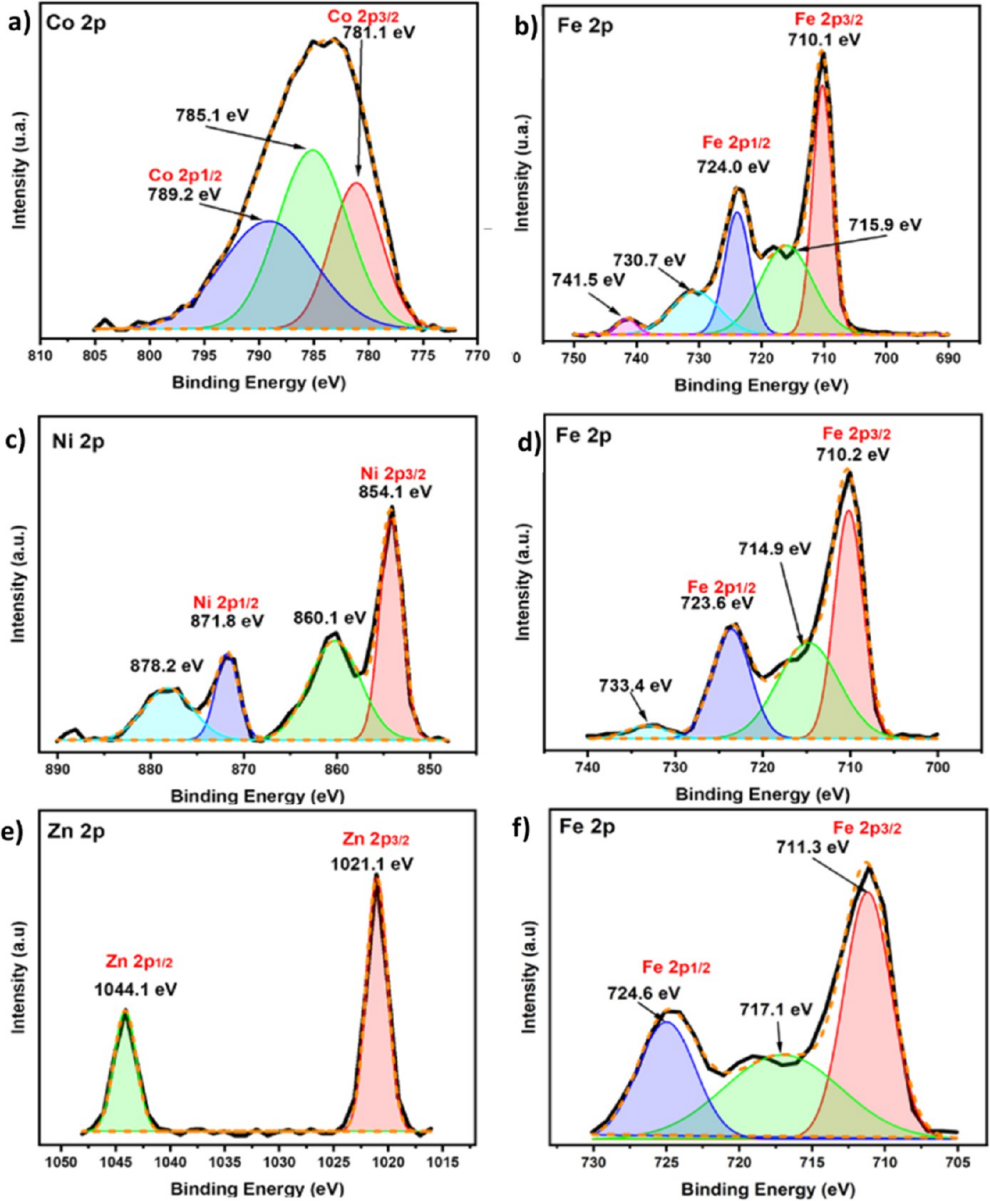
X-ray photoelectron spectra of the samples: **CQD@CoFe**_**2**_**O**_**4**_: (a) Co 2p and (b) Fe 2p; **CQD@NiFe**_**2**_**O**_**4**_: (c) Ni 2p and (d) Fe 2p; **CQD@ZnFe**_**2**_**O**_**4**_: (e) Zn 2p and (f) Fe 2p.

X-ray photoelectron spectroscopy (XPS) was used to study the elemental composition and oxidation state of the metals in the samples. The peak binding energy and full width half maxima (fwhm) (Table 2 and Figures 7 and S4) were analyzed and showed that C, O, and Fe are commonly presented in all the samples in addition to Co^2+^ for CQD@CoFe_2_O_4,_ Ni^2+^ for CQD@NiFe_2_O_4,_ and Zn^2+^ for CQD@ZnFe_2_O_4._ For instance, for CQD@CoFe_2_O_4_, the peaks related to Co 2p, such as Co 2p_3/2_ (781 eV) and Co 2p_1/2_ (789.2 eV) were detected in the high-resolution XPS and the expected satellite peak was also observed at 785.1 eV; additionally, the two signals corresponding to Fe 2p (Fe^3+^), namely, Fe 2p_3/2_ at 710.1 eV and Fe 2p_1/2_ at 724.0 eV were seen. The appearance of the signals matching to Co 2p_3/2_ and Fe 2p_3/2_ confirms the existence of Co^2+^ and Fe^3+^ in the samples, agreeing with previous reports, and it emphasizes that Co^2+^ and Fe^3+^ ions are typically occupied in the tetrahedral and octahedral sites, respectively.^103,104^ In the case of CQD@NiFe_2_O_4_, we observed the presence of Fe 2p and Ni 2p. Specifically, we noted four peaks, of which two were assigned to Ni 2p_3/2_ (854.1 eV) and Ni 2p_1/2_ (871.8 eV), and the remaining two are satellite peaks (860.1 and 878.2 eV). Furthermore, two peaks of the 2p_3/2_ and 2p_1/2_ orbitals (710.2 and 723.6 eV) are assigned to Fe 2p (Fe^3+^). The observed Ni^2+^ signals are consistent with the reported works that confirm the substitution of Ni ions in octahedral sites, enhancing the electronic interaction with Fe^3+^ ions.^105,106^ The XPS of CQD@ZnFe_2_O_4_ displayed peaks at 1044.01 and 1021.10 eV, corresponding to the Zn 2p_1/2_ and 2p_3/2_ states, respectively, indicating the presence of Zn in the +2-oxidation state. Additionally, signals for Fe^3+^ were observed at 711.08 eV for 2p_1/2_ and 711.30 eV for 2p_3/2_. For all the samples, the satellite signals for Fe 2p were consistently detected, specifically at 715.9, 730.7, and 741.5 eV for CQD@CoFe_2_O_4_; 714.9 and 733.4 eV for CQD@NiFe_2_O_4_; and at 717.10 eV for CQD@ZnFe_2_O_4_. These satellite peaks are grown from the electronic transitions within the d-orbitals.^106,107^ In the XPS spectra of all CQD@MFe_2_O_4_ samples, the deconvolution of O 1s at 529.80 eV indicates the adsorption of OH on the material’s surface.^108−110^ The O 1s peaks at 529.2 and 530 eV in the deconvolution peaks for CQD@CoFe_2_O_4_ correspond to the Co–O–Fe (metal–oxygen bond),^111−113^ indicating the distribution of cations in both octahedral and tetrahedral sites within the spinel ferrite structure.^114^ In the case of CQD@NiFe_2_O_4_, the three deconvolution peaks for O 1s at 529.0, 530.2, and 533.4 eV are attributed to the bonding of C–OH/C–O–C with Fe and Ni ions, confirming the existence of Ni/Fe–O bonds.^105,115^ Likewise, for CQD@ZnFe_2_O_4_, the O 1s signals at 531.40 and 529.80 eV representing the reticular oxygen bonded to Zn and Fe indicate the presence of Zn–O and Fe–O bonds.

**Table 2:** XPS Binding Energy Data (eV) for CQD@MFe_2_O_4_ (M = Co^2+^, Ni^2+^, and Zn^2+^)

sample	element transition	binding energy (*E*_b_)	standard binding energy data (*E*_std_) reported^117−119^	energy shift (*E*_b_–*E*_std_)	fwhm	ref
CQD@CoFe_2_O_4_	C 1s	282.8	283.9	–1.1	3.49	(120)
	O 1s	529.2	529.7	–0.5	2.03	
	Fe 2p_1/2_	724.0	723.5	0.5	4.62	
	Fe 2p_3/2_	710.1	710.5	–0.4	3.71	
	Co 2p_1/2_	789.2	793.3	–4.1	10.27	
	Co 2p_3/2_	781.0	780.9	0.1	5.51	
CQD@NiFe_2_O_4_	C 1s	284.1	283.9	0.2	2.10	(121)
	O 1s	529.1	529.7	–0.6	2.16	
	Fe 2p_1/2_	723.6	723.5	0.1	8.23	
	Fe 2p_3/2_	710.2	710.5	–0.3	3.64	
	Ni 2p_1/2_	871.8	870.18	1.6	2.95	
	Ni 2p_3/2_	854.1	853.8	0.3	2.84	
CQD@ZnFe_2_O_4_	C 1s	285.1	283.9	1.2	2.31	(122)
	O 1s	529.8	529.7	0.1	2.70	
	Fe 2p_1/2_	724.6	723.5	1.1	4.98	
	Fe 2p_3/2_	711.3	710.5	0.8	3.86	
	Zn 2p_1/2_	1044	1044.7	–0.7	2.64	
	Zn 2p_3/2_	1021.1	1021.8	–0.7	2.25	

Similarly, the deconvolution of C 1s was also observed in all samples, as CQDs contain carbon (398.08 eV) and nitrogen (399.08 eV), resulting in an energy difference of 1.0 eV between C 1s with a value of 395.08 eV, conforming to the deposition of CQDs on the ferrites. For instance, in CQD@CoFe_2_O_4_, C 1s was deconvoluted into three peaks, corresponding to 282.8 eV for the C–C bond, 284.0 eV for the C=C bond, and 286.6 eV for the C–O bond. For CQD@NiFe_2_O_4_, four distinct peaks (284.1 285.1, 286.1, and 288.1 eV) assigned to C=C/C–C, C–N, C–O, and C=O were detected, respectively. As for CQD@ZnFe_2_O_4_, the deconvolution peaks for C 1s show the existence of C–C (285.0 eV), C–O or C–N (287.6 eV), and C=O (291.7 eV) bonds.^116^ The deconvolutions of C 1s and O 1s highlight the role of functional groups in CQDs in enhancing the electronic interaction with ferrites.

### 2.9 Solid-State Reflectance Spectra

**Figure 8:**
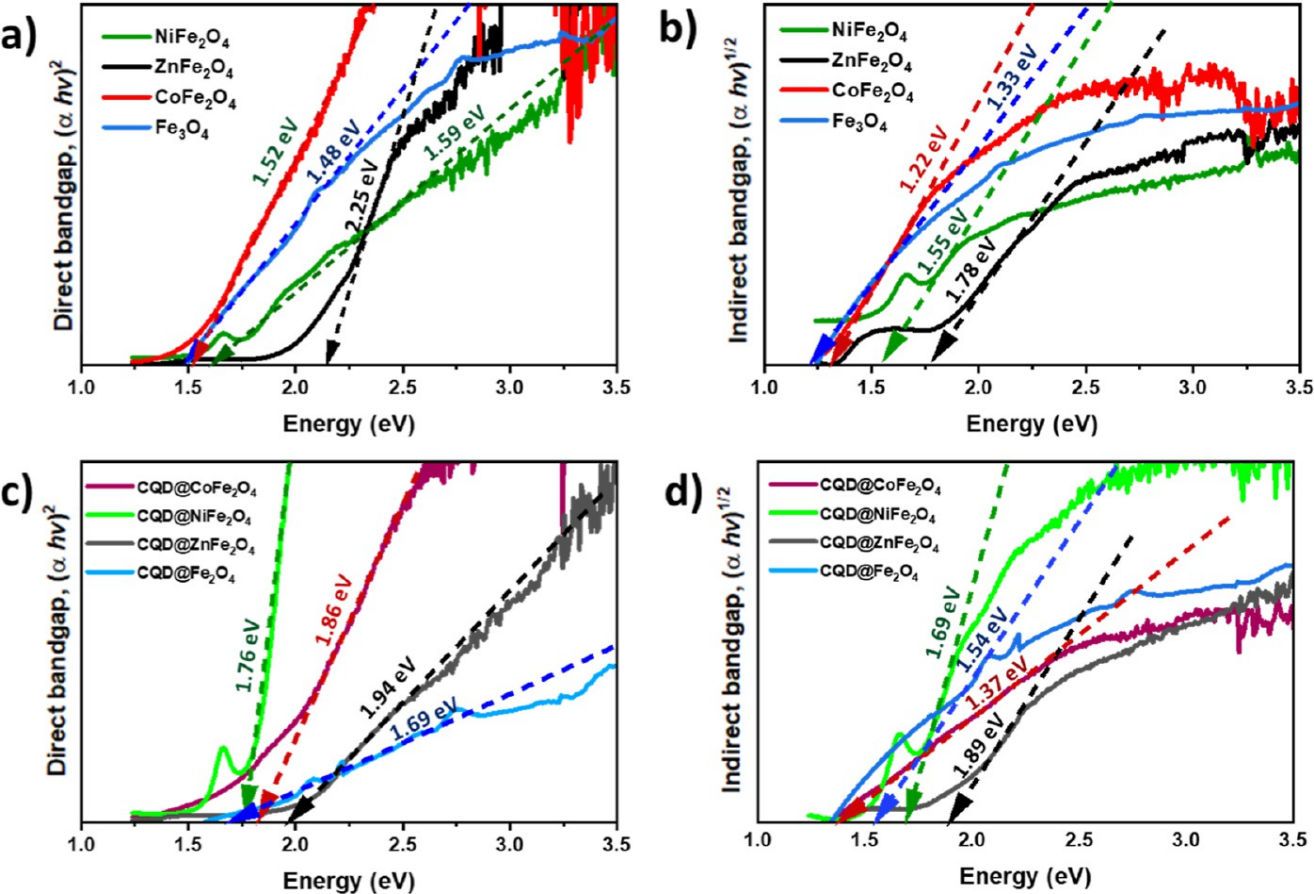
Tauc plots: (a) MFe_2_O_4_ (M = Fe ^3+^, Co^2+^, Ni^2+^, Zn^2+^) (direct); (b) MFe_2_O_4_ (M = Fe ^3+^, Co^2+^, Ni^2+^, Zn^2+^) (indirect); (c) MFe_2_O_4_ CQD (M = Fe ^3+^, Co^2+^, Ni^2+^, Zn^2+^) (direct); (d) MFe_2_O_4_ CQD (M = Fe ^3+^, Co^2+^, Ni^2+^, Zn^2+^) (indirect).

Electronic absorption and emission spectra for CQD were recorded and then analyzed, and the results show the absorption band at 360 nm corresponding to the π–π* transition.^79,123^ The fluorescence emission of CQD at 430 nm after the excitation at 310 nm was seen.^123^ The UV–vis solid reflectance spectra were also recorded for CQD@CoFe_2_O_4,_ CQD@NiFe_2_O_4,_ and CQD@ZnFe_2_O_4_ and calculated the band gap energy (*E*_g_) (Figure 8) through the Tauc plot extrapolating to *X* axis using the eq 2(124)
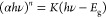
2*h*ν = energy of the incident photon, α = absorption coefficient, *K* = energy constant, and *n* = representation of the transition nature, with value of n = 2. The value of *E*_g_ is presented in electron Volts [eV] (*J* = 1.602 × 10^–19^ eV m) (Table 3 and Figure 8).

**Table 3:** Band Gap Energy Data of the Samples Was Calculated Using the Tauc Method

sample	direct bandgap (α*h*ν)^2^ [eV]	indirect bandgap (α*h*ν)^1/2^ [eV]	*E*_g_ (eV) ref. value	ref
Fe_3_O_4_	1.48	1.33	∼2.40–1.90	(127)
CoFe_2_O_4_	1.52	1.22	1.17–1.34	(128)
NiFe_2_O_4_	1.59	1.55	2.19	(129)
ZnFe_2_O_4_	2.25	1.78	∼1.90	(130)
CQD@Fe_3_O_4_	1.69	1.54	∼2.40–1.90	(127)
CQD@CoFe_2_O_4_	1.86	1.37	1.17–1.34	(128)
CQD@NiFe_2_O_4_	1.76	1.69	2.19	(129)
CQD@ZnFe_2_O_4_	1.94	1.89	∼1.90	(130)

The reddish-brown color of Fe_3_O_4_ (also known as magnetite) comes from ligand metal charge transfer, as both spin and d–d transitions are typically unlikely. However, in cases where magnetic coupling occurs between neighboring Fe^3+^ ions at an appropriate distance, these d–d transitions (^6^A_1_ → ^4^T_1_, ^6^A_1_ → ^4^T_2_, and ^6^A_1_ → ^4^E) become highly feasible. In the case of CoFe_2_O_4_, NiFe_2_O_4_, and ZnFe_2_O_4_, a strong peak is observed at 468, 516, 495, and 501 nm, which corresponds to the electronic transition from the VB (coming from the O 2p orbital) to the CB (involving the 3d orbital of Fe). VB stands for the highest occupied molecular orbitals formed by the overlap of the 2p states (oxygen ions) with some mixing of 3d states (Fe ions). At the same time, the CB represents the lowest unoccupied energy molecular orbitals. The absorption of visible light by bare ferrites is minimal, but for MFe_2_O_3_ NPs, a new band appears around 500 nm. This change is attributed to the structural configuration of the ferrites, which is influenced by the occupation percentage of metal ions, especially Co^2+^, Ni^2+^, Zn^2+^, and Fe^2+^, in octahedral and tetrahedral geometry. Fe^3+^ is positioned at the Oh site in the spinel configuration, while M^2+^ prefers the Td site, leading to different electronic arrangements. The bandgap energy corresponding to the absorption band was calculated using the Tauc method to determine both direct and indirect band gap energies.^125,126^ The DRS spectra were used to determine an absorption coefficient as α = *A*(*h*ν – *E*_g_)^2^/λ for direct (allowed) and α = *A*(*h*ν – *E*_g_)^1/2^/λ for indirect (allowed) (α = absorption coefficient; *A* = absorption constant for indirect transitions depending on the transition probability). The tangent line was drawn to point to the bandgap energy after the extrapolation of the *y*-axis against the photon energy *x*-axis (*h*ν); the Tauc’s plot [extrapolation of (α*h*ν)^1/2^ vs photon energy] results were: 1.57 eV for CQDs, 1.48 eV for Fe_3_O_4_, 1.52 eV for CoFe_2_O_4_, 1.1.59 eV for NiFe_2_O_4_, 2.25 eV for ZnFe_2_O_4_, 1.86 eV for CQD@CoFe_2_O_4_, 1.76 eV for CQD@NiFe_2_O_4_, and 1.94 eV for CQD@ZnFe_2_O_4_, and 1.69 eV for CQD@Fe_3_O_4_. The bandgap energy values are relatively consistent with those reported in the literature (Table 3). Notably, ferrite samples with CQD deposition show a significant reduction in band gap energy, as the CQD acts as an electron donor/acceptor to/from the CB of the ferrites. Additionally, the grain size of the samples notably contributes to the decrease in values, except for CQD@ZnFe_2_O_4_, where the bandgap energy value increases.

### 2.10 Brunauer–Emmett–Teller

The textural characterization of the samples was studied and performed on a Micromeritics ASAP 2020 automatic analyzer, operating at liquid nitrogen temperature (−196 °C). The samples were completely degassed (*p* < 10^–1^ Pa) at 200 °C for 6 h, and then the experiments were carried out by the Brunauer–Emmett–Teller (BET) method (SBET). The results (Table 4 and Figure S5) revealed that the incorporation of CQD into zinc ferrite (CQD@ZnFe_2_O_4_) significantly improved its textural properties, increasing the specific surface area from 8.39 m^2^/g for ZnFe_2_O_4_ to 8.66 m^2^/g for CQD@ZnFe_2_O_4_. Similarly, the total pore volume (*V*_p_, at a relative pressure of 0.98) increased from 0.0538 to 0.0621 cm^3^/g. The average pore diameter analyzed by the BJH method was found to be increased from 256.3 Å for ZnFe_2_O_4_ to 286.8 Å for CQD@ZnFe_2_O_4_, suggesting that it has enhanced the pore accessibility because of CQD incorporation which not only modifies the porous structure but also increases the possibility of the CO_2_ adsorption by providing additional active sites. The same trend data were established for NiFe_2_O_4_, CQD@NiFe_2_O_4_, CoFe_2_O_4,_ and CQD@CoFe_2_O_4_, seeing that the surface area (7.65 m^2^/g) and average pore diameter (133.3 Å) of NiFe_2_O_4_ are increased to 6.20 m^2^/g and 150.4 Å for CQD@NiFe_2_O_4_, respectively, although a total pore volume was slightly decreased to 0.0233 from 0.0255 cm^3^/g. For CoFe_2_O_4_ and CQD@1CoFe_2_O_4_, the data were as follows: specific surface area: 4.68 and 5.49 m^2^/g; total pore volume: 0.0228 and 0.0240 cm^3^/g; average pore diameter: 195.1 and 174.2 Å, respectively, showing that the textural characterization of the samples was significantly improved if CQDs were added to metal ferrites, particularly, specific surface area and pore accessibility.

**Table 4:** BET Results of MFe_2_O_4_ and CQD@MFe_2_O_4_

sample	BET surface area (m^2^/g)	total pore volume (cm^3^/g)	average pore diameter (Å)
CoFe_2_O_4_	4.68	0.0228	195.1
NiFe_2_O_4_	7.65	0.0255	133.3
ZnFe_2_O_4_	8.39	0.0538	256.3
CQD@CoFe_2_O_4_	5.49	0.0240	174.2
CQD@NiFe_2_O_4_	6.20	0.0233	150.4
CQD@ZnFe_2_O_4_	8.66	0.0621	286.8

### 2.11 CO_2_ Adsorption

**Figure 9:**
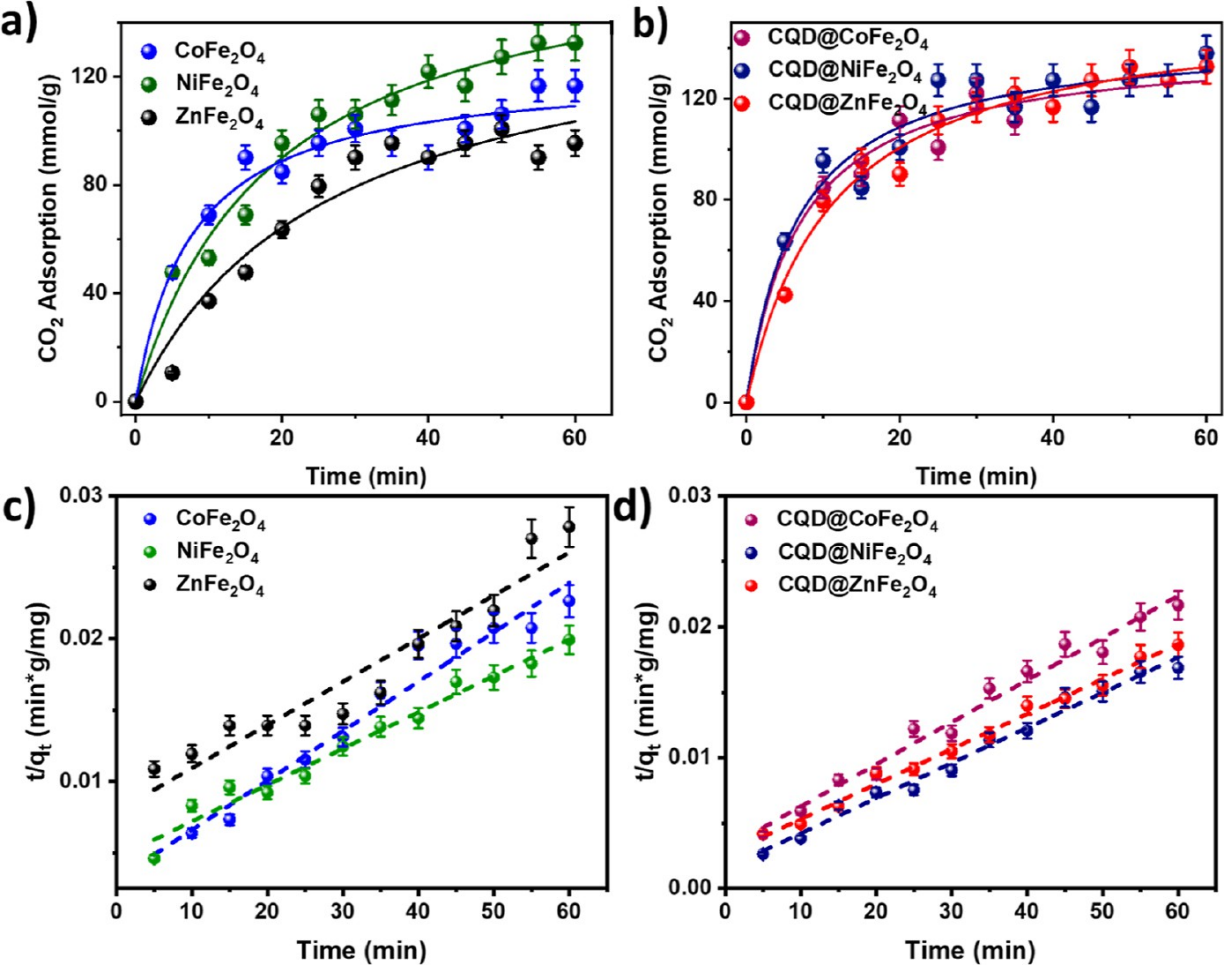
CO_2_ adsorption with MFe_2_O_4_: (a) Langmuir adsorption of CO_2_ with MFe_2_O_4_; (b) CO_2_ adsorption percentage for CQD@MFe_2_O_4_; (c) pseudo-second-order CO_2_ adsorption with MFe_2_O_4_ kinetics; and (d) pseudo-second-order adsorption CO_2_ kinetics for CQD@MFe_2_O_4_.

The CO_2_ adsorption was performed using MFe_2_O_4_ and CQD@MFe_2_O_4_; before the experiment, the ferrite samples underwent thermal treatment at 200 °C for 3.0 h for degasification. The samples were placed in glass capsules within a closed chamber operating at room temperature and pressure conditions (1.0 atm and ∼25 °C). The CO_2_ that flew through the sample was 20 mL/min, and the CO_2_ concentration in the outlet was monitored over 60 min (Figure 9a,b). The adsorption data were plotted against time, demonstrating significant CO_2_ adsorption in the samples. For instance, the sample weight of CoFe_2_O_4_ increased from 0.0503 to 0.0518 g after 60 min of the CO_2_ treatment with the sample. Although the general trend was similar for all samples, CQD@NiFe_2_O_4_ exhibited superior CO_2_ adsorption at 96.77 ± 0.82%. The plotted data closely aligned with the Langmuir model, excellent fitting (*R*^2^ > 0.96) into pseudo-second-order kinetics^131^ (Table 5 and Figure 9c,d). The experimental results revealed that the pseudo-first-order model is unsuitable for the studied systems, as the coefficients of determination (*R*^2^) were consistently lower than 0.7, indicating a poor fit and limiting the behavior of first-order adsorption kinetics. The above description of adsorption kinetics accurately provides a fundamental tool for optimizing the design and use of adsorbents in practical applications.^132,133^ On the other hand, the Langmuir model reinforces the understanding of adsorption equilibrium by describing monolayer behavior and validating the specificity and uniformity of the active sites.

**Table 5:** CO_2_ Adsorption Data of Ferrite Samples (CO_2_ Gas Flow 20 mL/min at 1.0 atm in ∼25 °C)^a^

samples	adsorption (mmol/g) (%)	Langmuir fit (*R*^2^)	*t*/*q*_*t*_ (×10^–4^) (min·g/mg)	kinetic constant (*k*_2_) [g/(mg·min)]	kinetic fit (*R*^2^)
CoFe_2_O_4_	2.90 (84.6 ± 1.2)	0.961	3.461 ± 1.008	4.11	0.966
NiFe_2_O_4_	3.10 (89.3 ± 1.0)	0.978	2.548 ± 0.793	4.33	0.972
ZnFe_2_O_4_	2.62 (72.0 ± 0.9)	0.947	3.022 ± 0.976	7.12	0.910
CQD@CoFe_2_O_4_	3.37 (95.8 ± 1.2)	0.966	2.678 ± 0.936	4.86	0.978
CQD@NiFe_2_O_4_	4.33 (96.8 ± 0.8)	0.958	2.688 ± 0.759	2.94	0.984
CQD@ZnFe_2_O_4_	3.52 (93.1 ± 0.6)	0.985	3.219 ± 3.440	2.99	0.991

a Note: (mmol/g) (%) = concentration and percentage of CO_2_ absorbed, respectively; *t*/*q*_*t*_ = time normalized by the amount adsorbed.

**Figure 10:**
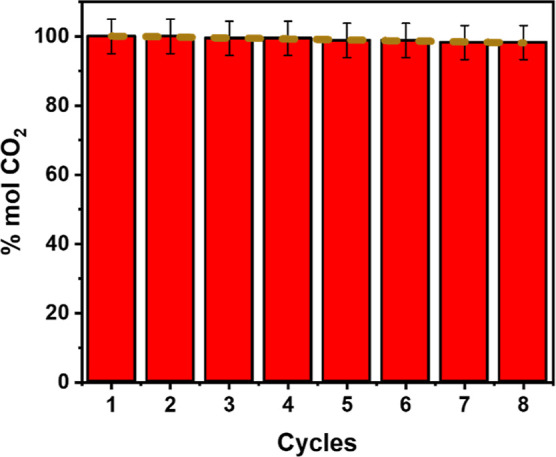
Cycles of CO_2_ adsorption by CQD@NiFe_2_O_4_ flow gas 20 mL/min, at ∼25 °C, 1 atm.

After subjecting CQD@NiFe_2_O_4_ to thermal treatment at 200 °C for 3 h, adsorption cycles are performed, as illustrated in Figure 10. The initial adsorption conditions were provided in the adsorption chamber, and results were recorded after 60 min and repeated eight times. Figure 10 displays the percentage of adsorption efficiency over the number of desorption cycles. The first two cycles maintained an adsorption efficiency of 100%. There was a slight drop to 99.42% in the third and fourth cycles. Subsequently, cycles 5 and 6 showed around a 1% drop in adsorption efficiency, while cycles 7 and 8 exhibited similar adsorption rates of 98.82% and 98.83%, respectively.

In the previous works (Table 6), several materials have been explored for the adsorption of CO_2_, and the present results are comparable with the reported values at room temperature although KOH/carbonized pollen and NbOFFIVE-1-Ni (MOF with niobium (Nb) and a specific structure within the OFFIVE family) adsorb more than 10 mmol/g and BaNi_2_Fe_16_O_27_ has the ability to adsorb around 1.0 mmol/g.

**Table 6:** Different Adsorbents for the Adsorption of CO_2_ from the Literature^a^

material	CO_2_ adsorption capacity (mmol/g)	adsorption conditions	ref
zeolites KX, MgX	4.80	25 °C, 1.0 atm	(134)
CaLTA zeolite	3.30	25 °C, 1.0 atm	(135)
4A-zeolite	1.40	25 °C, 1.0 atm	(136)
Mg-MOF-74	8.00	25 °C, 1.0 bar	(137)
Mg2(dobdc)	6.10	25 °C, 150 mbar	(138)
NbOFFIVE-1-Ni	10.10	298 K, 100 mbar	(66)
BaNi_2_Fe_16_O_27_	0.84	30 °C	(62)
KOH/carbonized pollen	33.8	25 °C, 1.0 bar	(67)
2.5 wt % SiO_2_–K.WT	2.53	25 °C, 1.0 bar	(139)
Pt–Ca/Al_2_O_3_	1.07	300 °C, 100 Pa	(140)
ZF/rGO-MEA30	9.32	25 °C, 1.0 bar	(61)

a Note: KX, MgX where X = faujasite zeolite with low silica to alumina ratio; CaLTA = Clinoptilolite and the LTA (Lynde-Type A); 4A = zeolite with 4 Å pores; dobdc = 2,5-dioxide-1,4-benzenedicarboxylate; NbOFFIVE = MOF with niobium (Nb) and a specific structure within the OFFIVE family; waste tea = WT; ZF/rGO-MEA30 = zinc ferrite/amine-functionalized reduced graphene oxide nanocomposites.

### 2.12 Electrochemical Sensing of CO_2_

**Figure 11:**
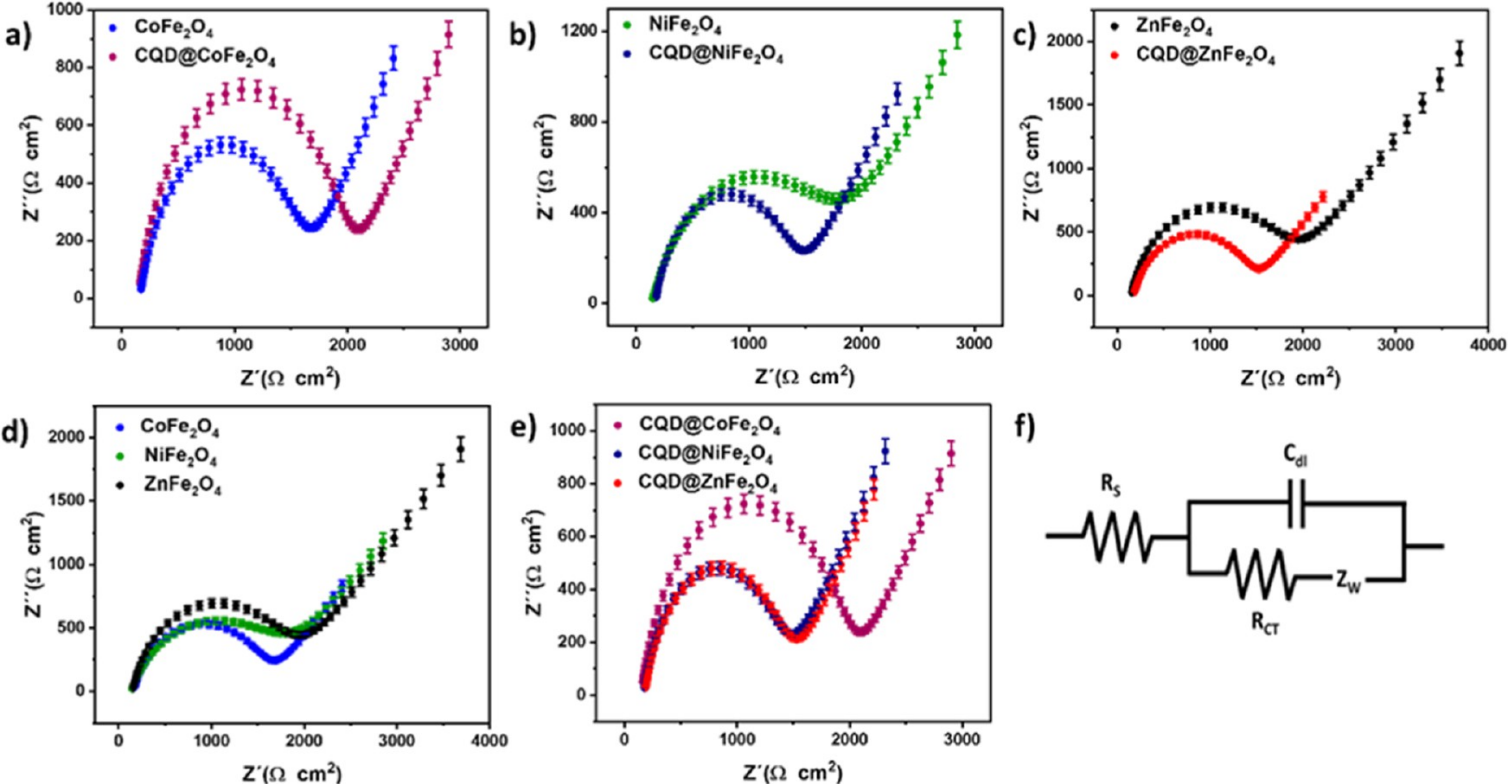
Electrochemical impedance Nyquist diagrams: (a) CPE/CoFe_2_O_4_ and CPE/CQD@CoFe_2_O_4_, (b) CPE/NiFe_2_O_4_ and CPE/CQD@NiFe_2_O_4_, (c) CPE/ZnFe_2_O_4_ and CPE/CQD@ZnFe_2_O_4_; (d) comparison of bare ferrites; and (e) comparison of doped ferrites. [Fe(CN)_6_]^3–^/[Fe(CN)_6_]^4–^ (5.0 mM) in BRB (pH 7.0, 0.04 M) was used as a substrate; (f) Randles equivalent circuit.

The electrochemical impedance behaviors of the samples were analyzed using a potentiostat/galvanostat (ACM Gill AC) under open circuit conditions, with a frequency range of 10 kHz to 0.1 Hz and an alternating current voltage amplitude of 10 mV. The equipment was controlled by a computer running ACM software. Impedance data were collected using an electrochemical cell with a three-electrode configuration, where the working electrode (WE) was carbon paste electrodes (CPE)/CoFe_2_O_4_, CPE/NiFe_2_O_4_, CPE/Zn Fe_2_O_4_, CPE/CQD@CoFe_2_O_4_, CPE/CQD@NiFe_2_O_4_, or CPE/CQD@ZnFe_2_O_4_. The electrochemical impedance spectroscopy (EIS) studies were first performed using [Fe(CN)_6_]^3–^ /[Fe(CN)_6_]^4–^ (5.0 mM) in a Britton–Robinson buffer (BRB)solution (pH 7, 0.04 M) to assess whether the results conformed to the simple Randles equivalent circuit (Figure 11), incorporating solution resistance (*R*_s_), capacitance (CPE), charge transfer resistance (*R*_CT_), and the Warburg impedance (*W*_0_). Nyquist diagrams were constructed based on the impedance data obtained from the samples, taking into account the phase angle of current versus voltage. The data suggest a linear relationship between Z″ (ohm) and Z′ (ohm) at low frequency, indicating a mass transfer effect as the resistance data closely align with a simple Randles equivalence circuit. However, at higher frequencies, the semicircular loop diameter significantly increases, suggesting that the charge transfer resistance (*R*_CT_) at the electrode boundaries (interface) is much higher than at low frequencies (Table S5). Compared to CPE/CQD@MFe_2_O_4_ (lower *R*_CT_), CPE/MFe_2_O_4_ exhibits a larger semicircle diameter and a greater *R*_CT_ (Figure 11a–c). For example, the resistance per area was 1853 Ω cm^2^ for CPE/CoFe_2_O_4_ (Figure 11d), whereas it decreased to 1652 Ω cm^2^ for CPE/NiFe_2_O_4_ and 1672 Ω cm^2^ for CPE/ZnFe_2_O_4_. The transfer of electrons for CPE/CoFe_2_O_4_ requires greater resistance and energy than those for CPE/NiFe_2_O_4_ or CPE/ZnFe_2_O_4_. This is evident from the trend observed: 1440 Ω cm^2^ (CQD@CoFe_2_O_4_) > 1211 Ω cm^2^ (CPE/CQD@NiFe_2_O_4_) > 1277 Ω cm^2^ (CPE/CQD@ZnFe_2_O_4_) (Figure 11e), representing the Randles equivalent circuit (Figure 11f). CPE/CQD@MFe_2_O_4_ demonstrates improved electron transfer with lower resistance due to the composite nature of the sample (CQDs with MFe_2_O_4_).

**Figure 12:**
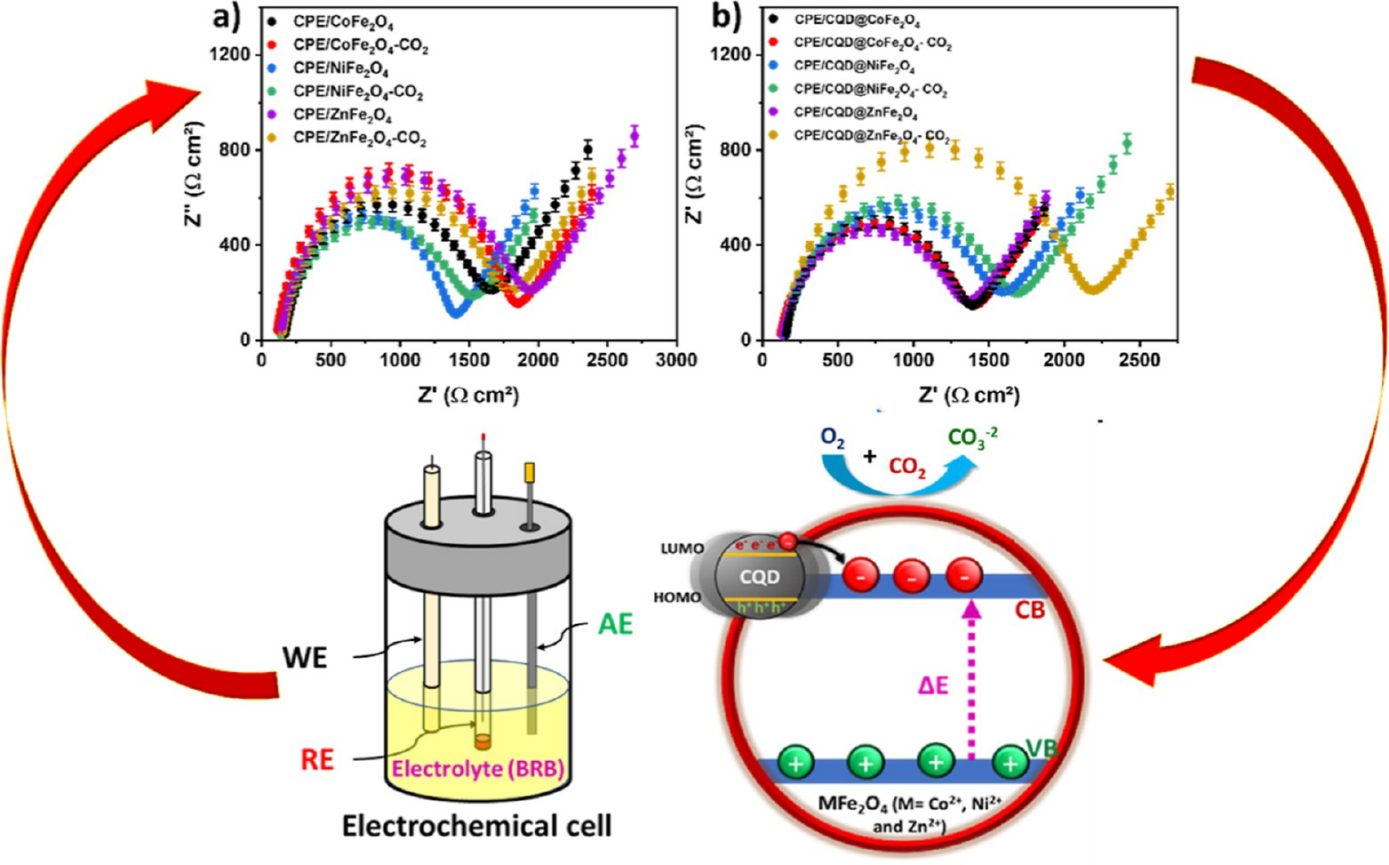
Electrochemical impedance Nyquist diagrams of the sample treated with CO_2_: (a) MFe_2_O_4_ and (b) CQD@MFe_2_O_4_ (M = Co^2+^, Ni^2+^, and Zn^2+^).

The CO_2_ capture of the samples was analyzed by using the experimental setup described above. CO_2_ flow (20 mL/min) was maintained for 60 min. The CO_2_-treated sample served as the WE, and impedance data were collected to assess the adsorption of CO_2_ on the sample surface and understand its impact on the electrode–solution interface. The results indicate a notable change in the *R*_CT_ for the CO_2_-treated sample. Specifically, the resistance was lower (1163 ± 14.27 Ω cm^2^) for CQD@ZnFe_2_O_4_–CO_2_ as compared to 1567 ± 35.74 Ω cm^2^ for CQD@ZnFe_2_O_4_, suggesting that greater values of *R*_s_, *R*_CT_, τ, and W0 were obtained for the CO_2_-treated samples. This indicates slower electrochemical kinetics at the electrode than the samples not treated with CO_2_ (see Table 7 and Figure 12).

**Table 7:** Electrochemical Impedance Data of CPE/MFe_2_O_4_–CO_2_ and CPE/CQD@MFe_2_O_4_–CO_2_ (M = Co^2+^, Ni^2+^, and Zn^2+^)^a^

sample	*R*_S_ (Ω cm^2^)	*R*_CT_ (Ω cm^2^)	τ (×10^–3^ s)	*W*_0_
CPE/CoFe_2_O_4_–CO_2_	162.3	1408	3.75	631.7
CPE/NiFe_2_O_4_–CO_2_	130.4	1527	6.99	409.5
CPE/ZnFe_2_O_4_–CO_2_	141.2	1848	2.95	552.6
CPE/CQD@CoFe_2_O_4_–CO_2_	114.4	1219	3.41	402.4
CPE/CQD@NiFe_2_O_4_–CO_2_	141.9	1458	2.98	652.7
CPE/CQD@ZnFe_2_O_4_–CO_2_	143.6	1567	4.38	541.8

a Note: *R*_S_ = solution resistance; *R*_CT_ = charge transfer resistance; τ = relaxation time constant; *W*_0_ = Warburg impedance.

Upon adsorption of CO_2_ on MFe_2_O_4_ and CQD@MFe_2_O_4_ (M = Co^2+^, Ni^2+^, and Zn^2+^), oxygen species (O_2_, O^–^, and O^2–^) initiate a reaction with CO_2_ facilitated by taking electrons from the CB that leads to the formation of CO_3_^2–^, which then converted into carbonic acid in the solution. This significantly enhances the interaction of CO_2_ with the surface, driven by chemisorption, reducing impedance resistance. Additionally, the presence of oxygen species with electron-donating capabilities enhances the electron density in the VB. Furthermore, the porous nature of MFe_2_O_4_ and CQD@MFe_2_O_4_ (M = Co^2+^, Ni^2+^, and Zn^2+^) is improved by the deposition of metal ions, generating crystal defects at the M–Fe_2_O_4_ interface, thereby increasing oxygen vacancies. This facilitates the reaction of O_2_, O^–^, and O^2–^ with CO_2_ to form CO_3_^2–^ (eqs 3–6).
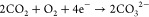
3
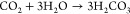
4
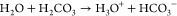
5
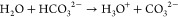
6

The low electrochemical resistance observed for the samples after CO_2_ treatment is attributed to the adsorption of CO_2_ on the surface, and it modifies the charge transfer characteristics of the system, which leads to the detection of CO_2_. EIS effectively recognizes the interaction of CO_2_ with the sample, sensing CO_2_. The results highlight the potentiality of the EIS technique in the practical application of advanced CO_2_ sensors, which are required to monitor the air quality, especially CO_2_ levels.^141,142^ Furthermore, the presence of CQD on the surface ferrite favors the CO_2_ chemisorption, allowing interaction with the CB electrons in the presence of dissolved oxygen or oxygen species (O_2_, O^–^, and O^2–^) to form carbonate (CO_3_)^2–^. The oxygen vacancies can be generated if Fe^3+^ is reduced by substituting metal ions such as Co^2+^, Ni^2+^, and Zn^2+^, increasing the *h*^+^ charge in the VB. These features generate more active sites that promote the chemical interaction with CO_2_. Moreover, the functional groups attached to CQDs can enhance CO_2_ adsorption because of greater interaction points.^143^ This synergistic effect significantly reduces electronic impedance, stabilizes intermediate species, and improves the catalytic CO_2_ capture efficiency.^144^ There is also a high possibility that if the thickness of the CO_3_^2–^ layer grows, the system may undergo a dis-adsorption of carbonate from the surface to the solution in forming carbonic acids (HCO_3_^–^ and H_2_CO_3_). This technique (circuiting it in the electrode attached to the portable potentiostat/galvanostat) can be easily applied in industrial CO_2_ gas emission, measuring the concentration of CO_2_ and capturing it by scaling up in large-scale of the samples.

**Figure 13:**
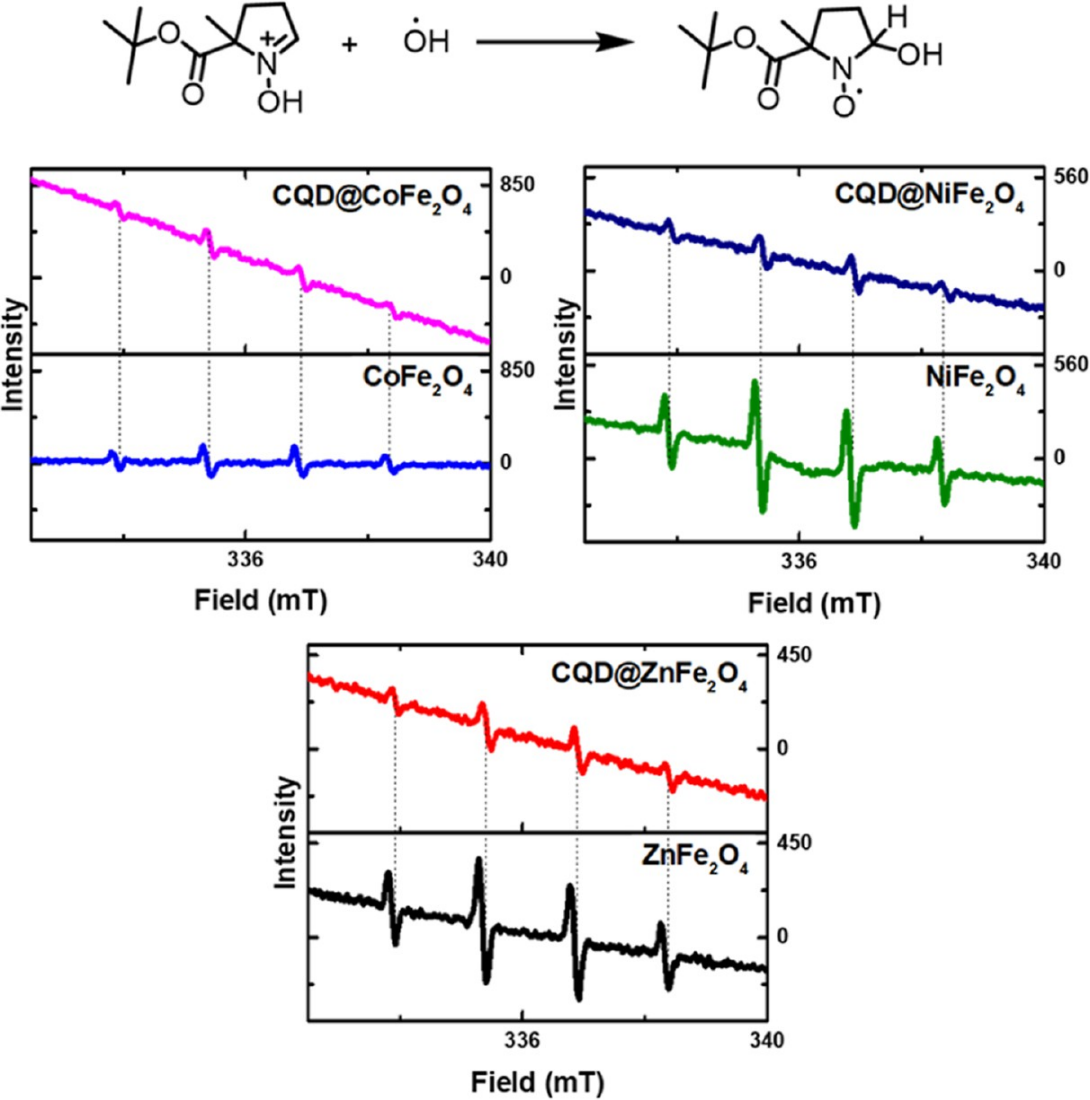
EPR of the samples: the spin-trapping agent BMPO with a hydroxyl radical forming the BMPO-radical adduct in aqueous suspensions of CQD@MFe_2_O_4_ and MFe_2_O_4_, M = Co^2+^, Ni^2+^, and Zn^2+^.

*Electron paramagnetic resonance (EPR) studies:* The production of radical species from the samples was detected by EPR; evidently, the positively charged holes produced in the VB of MFe_2_O_4_ NPs can react with H_2_O to create HO^•^ or the electron in the CB can react with O_2_ molecules to form oxygen species (O_2_, O^–^, and O^2–^) radicals.^145^ The formation of these radicals was identified by using EPR (Figure 13). In Figure 13a, it is shown that BMPO (cyclic nitrone spin trap) can combine with HO^•^ radicals to form a BMPO–OH radical adduct. This method enables the identification and characterization of short-lived radicals using EPR spectroscopy. The dashed lines (Figure 13b) represent the distinct hyperfine splitting of BMPO–OH. Furthermore, the peak intensities of the spectra follow the pattern MFe_2_O_4_ ≫ CQD@MFe_2_O_4_, except for CoFe_2_O_4_ and CQD@CoFe_2_O_4_, where the peak intensities remain constant.

### 2.13 Evaluation of the Industrial Feasibility

The production scalability of CQD@MFe_2_O_4_ (M = Co^2+^, Ni^2+^, and Zn^2+^) was analyzed using the mass intensity and the efficiency to scale up (eqs 7 and 8)^146,147^
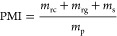
7
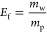
8

Process mass intensity (PMI) is related to the number of reagents (*m*_rc_), reactants (*m*_rg_), and solvents (*m*_s_) employed to obtain the final amount of product (*m*_p_). The preparation process minimizes the loss of reagents to maximize the formation of products, projecting an ideal PMI value of 1.0, and if PMI → ∞ (infinitive), the efficiency of the process can be decreased. In the early stage of the process, PMI can reach 500 kg/kg, although this value can be as low as 1.1 kg/kg for the petrochemical industry.^148,149^ In the early stage of the present work process, for the CQD production, PMI was 508.12; and for MFe_2_O_4_ (M = Co^2+^, Ni^2+^, and Zn^2+^), the calculated values resulted in 301.52, 288.87, and 286.85 kg/kg for Co, Ni, and Zn, ferrites, respectively. For CQD@MFe_2_O_4_, the prepared precursors (CQD and ferrites) were considered as reagents in the PMI calculation, resulting in 117.32 kg/kg. This indicates a higher efficiency in the formation of the final product.

The other evaluated indicator, Eco-factor (E-factor, *E*_f_), is associated with the amount of waste formed per unit during final product formation. This means that the scalability depends upon minimizing waste to get as low as *E*_f_ (*E*_f_ tends to approach 0) in an ideal process, suggesting that no waste is produced. So, all the factors are incorporated into the product manufacturing process.^150^ Typical values in specialty chemicals reach *E*_f_ > 500, with some cases above 2000 for the industries. In this process, waste generation is mainly related to the purification of the final product, where for CQD, *E*_f_ was 507.12, which corresponds to the degree of production or optimization of the materials. For the ferrites, *E*_f_ was 300.52 for CoFe_2_O_4_, 287.87 for NiFe_2_O_4_, and 285.85 for ZnFe_2_O_4_. Finally, for CQD@MFe_2_O_4_, *E*_f_ was 117.32, suggesting the high possibility of the scalability of this production stage as it is highly efficient for scaling up in terms of mass economy, although the production of CQDs and ferrites requires further optimization and minimization of reagents and waste.

### 2.14 DFT CO_2_ Adsorption Studies

The study optimizes a ferrite system comprising an M^2+^_32_Fe^3+^_64_O_128_ (M = Co^2+^, Ni^2+^, and Zn^2+^) and two CQD molecules, Co^2+^_32_Fe^3+^_64_O_128_, Ni^2+^_32_Fe^3+^_64_O_128_, and Zn^2+^_32_Fe^3+^_64_O_128_. Since the magnetic exchange coupling constant (*J*) is crucial for the ferrites,^151^ a suitable hybrid HSEH1PBE functional was chosen, as it can optimize the structure. For heavy atoms of the transition metals, LANL2DZ is being considered to determine the electronic properties.^152,153^ Thus, HSEH1PBE/LANL2DZ was chosen for the present study as reported previously.^154,155^ The study was used to determine the semiconductor nature, geometric characteristics (interband structure), and excitation states for interacting the ferrite system with CO_2_ molecules.

**Figure 14:**
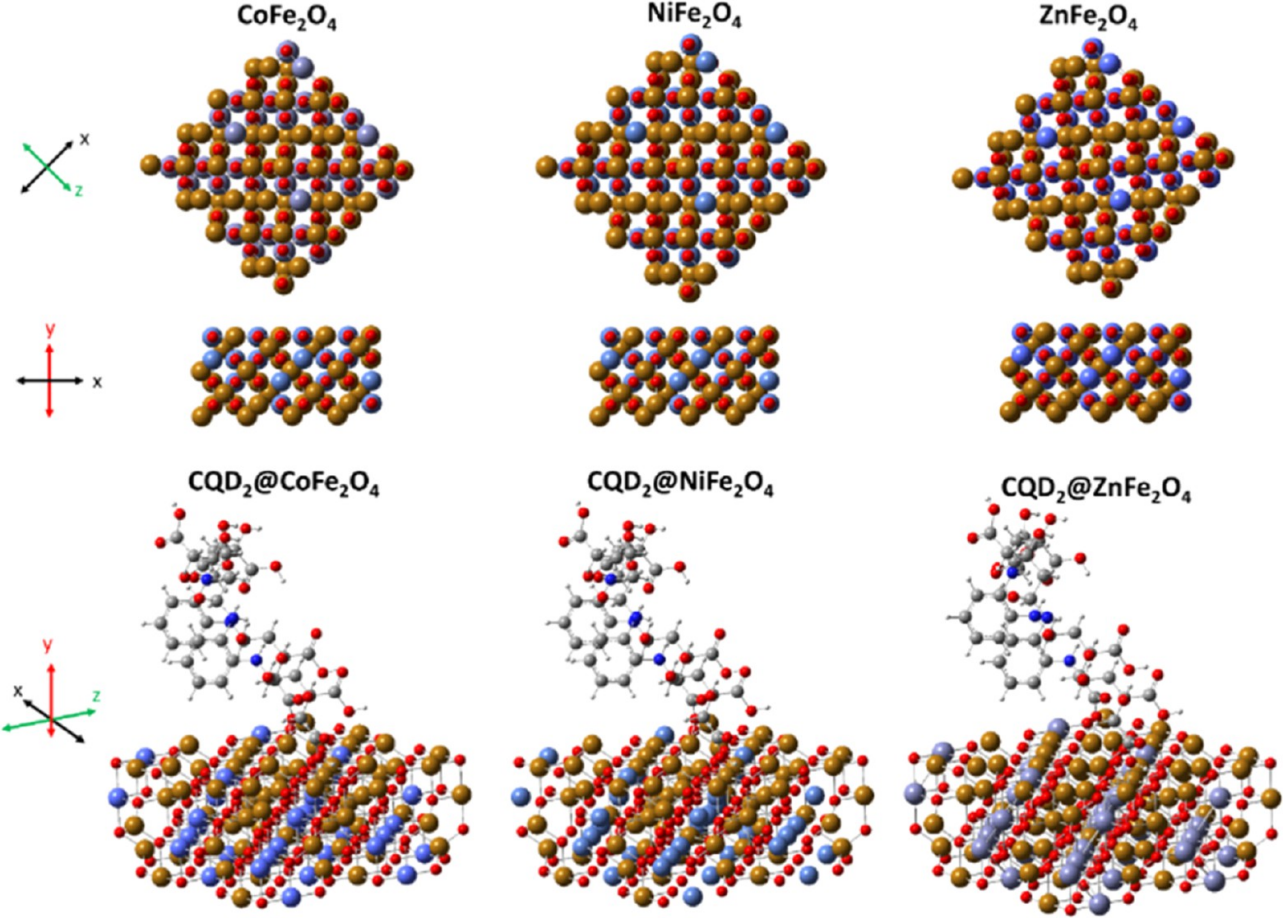
Optimized geometry of ferrites: CoFe_2_O_4_, NiFe_2_O_4_, and ZnFe_2_O_4_ and the interaction of CDs2 with CoFe_2_O_4_, NiFe_2_O_4_, and ZnFe_2_O_4_.

**Figure 15:**
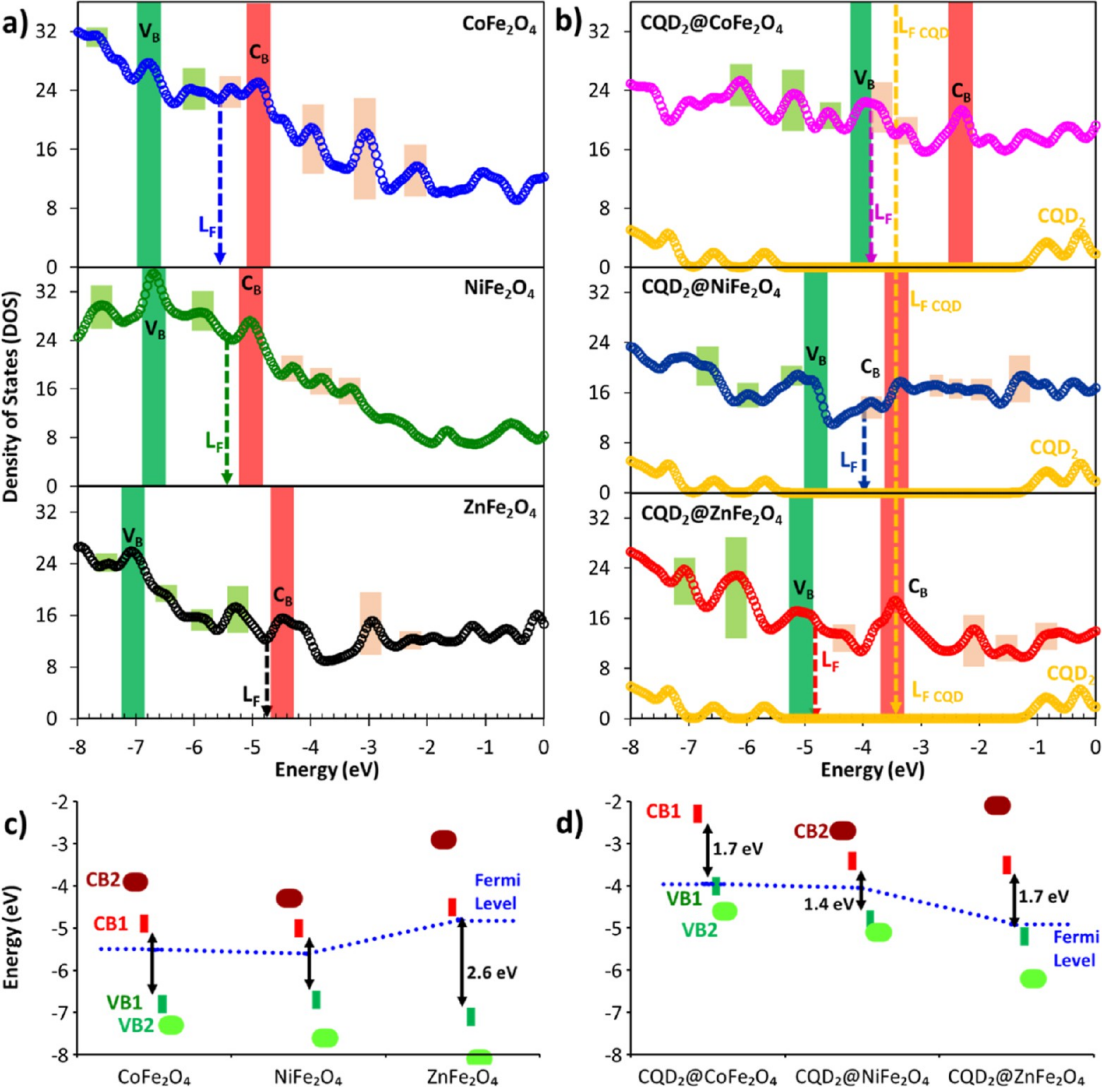
HSEPBE DOS profile at frontier bands: (a) metal ferrites of Co, Ni, and Zn; (b) changes in DOS profile upon the sensitization of the metal ferrites by CQD_2_; (c) Semiconductor band model of the metal ferrites and (d) semiconductor model of the metal ferrites with CQD_2_.

Optimized geometries of CoFe_2_O_4_, NiFe_2_O_4_, ZnFe_2_O_4_, and CQD2@CoFe_2_O_4_, CQD2@NiFe_2_O_4_, and CQD2@ZnFe_2_O_4_ are presented in Figure 14. The spinel ferrites consist of oxygen atoms closely packed with metal ions, thereby creating 96 interstitial sites, with 8 being tetrahedral sites occupied by M^2+^ (Co^2+^, Ni^2+^, and Zn^2+^), and 16 octahedral sites occupied by Fe^3+^ ions. The crystalline network parameters (*abc*) for CoFe_2_O_4_, NiFe_2_O_4_, and ZnFe_2_O_4_ were determined using DFT in HS1Pbe as follows: 10.56 × 8.28 × 16.56, 10.56 × 8.28 × 16.56, and 10.58 × 8.29 × 16.57 Å, respectively (Table S6). The DFT data show that CQD groups (CDS2) on the surface of MFe_2_O_4_ reduce the energy. Notably, the dipole moment (*p*) is greater for CoFe_2_O_4_ (5546.05 dy) compared to NiFe_2_O_4_ (2510.87 Dy) and ZnFe_2_O_4_ (2288.01 Dy), due to the difference in the mass ratio of Co^2+^ ions compared to Ni^2+^ and Zn^2+^. In the case of CDs2, the *p* value was 7.79 Dy, which decreased further when CDs2 adsorbed the ferrite systems, resulting in the formation of CDs2-CoFe_2_O_4_ (5243.28 Dy), CDs2-NiFe_2_O_4_ (1722.93 Dy), and CDs2-ZnFe_2_O_4_ (659.66 Dy). This suggests that C atoms in these systems tend to undergo charge transfer, leading to a decreased polarity. Using Perdew–Burke–Ernzerhof (PBE), we investigated the electronic properties of the ferrites and calculated the band gap energies using the density of states (DOS, Figure 15). The calculated band gap energies were as follows: 1.70 eV for CDs2-CoFe_2_O_4_, 1.70 eV for CDs2-NiFe_2_O_4_, and 1.40 eV for CDs2-ZnFe_2_O_4_ (Table S6), indicating a decrease in the band energy as compared to the experimental data of MFe_2_O_4_ and CQD@MFe_2_O_4_. CDs2 in ferrites modifies the electronic behavior, generating additional energy states in the occupied and unoccupied states that alter the band energy. All of the systems exhibited similar band energy, with four bands in the VB and two in the CB, resembling the behavior of magnetite. The introduction of CDs2 into ferrites also causes a significant change in the band positions. For CoFe_2_O_4_, the observed bands (−6.8 eV VB and −4.8 eV CB) shifted to −4.0 eV (VB) and −2.45 eV (CB) for CDs2-CoFe_2_O_4_. Similarly, the bands of NiFe_2_O_4_ at −6.8 eV (VB) and −5.0 eV (CB) shifted to −4.8 eV (VB) and −3.4 eV (CB) for CDs2-NiFe_2_O_4_. The same trend was observed for ZnFe_2_O_4_, and CDs2-ZnFe_2_O_4_, with band shifts from −7.2 eV (VB) and −4.6 eV (CB) to −5.2 eV (VB) and −3.6 eV (CB). These changes in the Fe–O interaction alter the occupied and unoccupied states, as shown in Figure 15. Finally, the DFT band gap energy is compared with the band gap energy obtained from the Tauc plot (Table S7).

**Figure 16:**
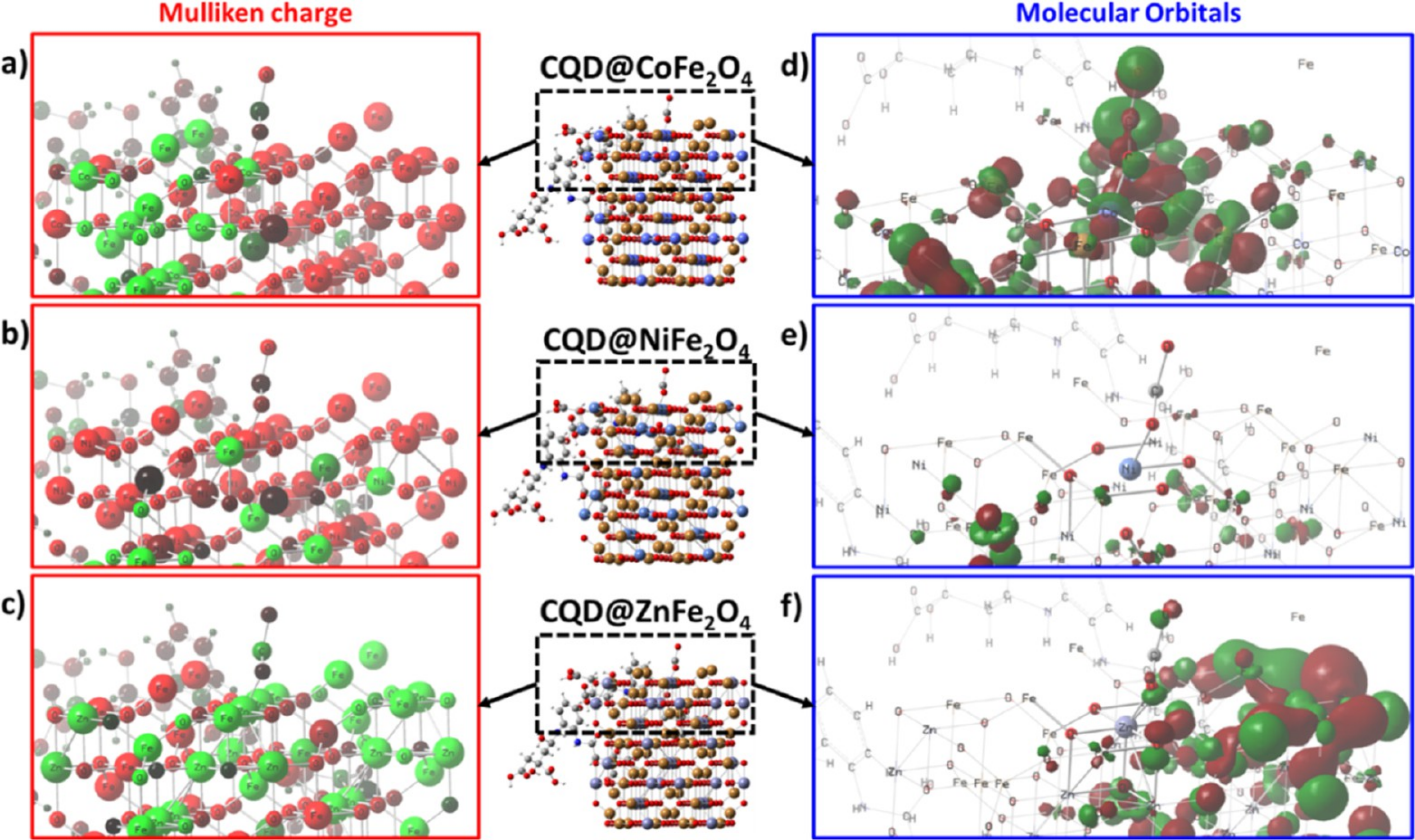
DFT study of CO_2_ adsorption onto CQDs doped ferrites. Mulliken charge: (a) CQD@CoFe_2_O_4_; (b) CQD@NiFe_2_O_4_; and (c) CQD@ZnFe_2_O_4_. MO mapping: (d) CQD@CoFe_2_O_4_; (e) CQD@NiFe_2_O_4_; and (f) CQD@ZnFe_2_O_4_.

The adsorption of CO_2_ on ferrites was studied by comparing the geometries and energies before and after carbon dioxide adsorption onto CQD@MFe_2_O_4_ (Figure S6). The results show that the adsorption of CO_2_ depends on the availability of active sites where the O=C=O molecule could be attached to the ferrites by either an O–M or C–O bond. The high electronegativity of O in carbon dioxide favors the interaction with metal (oxygen–metal), deforming the molecule’s symmetry’s linearity and leading to a subsequent C–O interaction to form carbonate. In this study, the proposed interaction occurs at the surface of tetrahedral A sites occupied by Co^2+^, Ni^2+^, or Zn^2+^. The relative charges at the MFe_2_O_4_ surface (Figure 16) show that CQD@CoFe_2_O_4_ (Figure 16a), the superficial Co^2+^, has a relatively positive charge allowing interaction with ^δ−^O in CO_2_. Furthermore, spin density mapping shows a highly conjugated system where the interaction would take place by overlapping of Co^2+^ d_*z*^2^_ with O^2–^ 2sp; even though octahedral Fe^3+^ are presented, the higher energy of d_*xyz*_ redirects CO_2_ bonding toward more available Co ions (Figure 16b).^156^ For CQD@NiFe_2_O_4_, it can be observed that the surface atoms have a relatively high negative charge, hindering the interaction with oxygen in CO_2_; moreover, spin density mapping shows very low density and minimal conjugation that would hinder Ni^2+^–O^2–^ bonds (Figure 16c,d). For CQD@ZnFe_2_O_4_, the positive charges at the surface of ferrites allow for the adsorption of CO_2_ molecules; however, the spin conjugation is weak, and Zn^2+^–O^2–^ interaction would occur by electrostatic charges and is supported by d_*xyz*_ metal ions. This interaction is therefore expected to be present but weak, which supports experimental findings where the strongest interaction was for Co materials followed by Zn and Ni ferrites.

**Figure 17:**
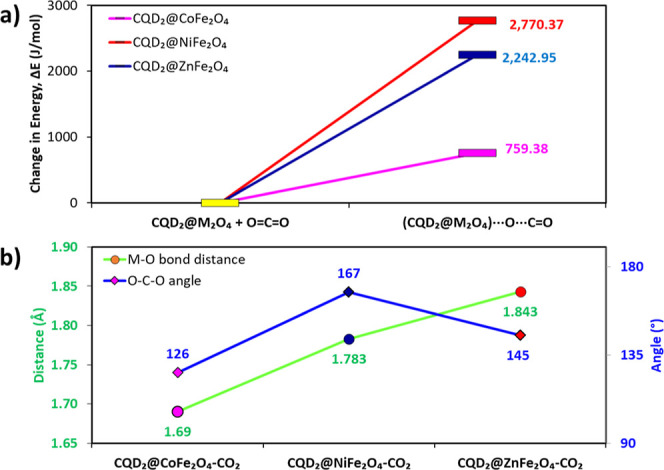
(a) Energy change observed for CQD@MFe_2_O_4_ after the interaction of carbon dioxide with the ferrites; (b) metal–oxygen bond distances and CO_2_ bond angles upon adsorption over studied CQD@MFe_2_O_4._.

The adsorption of CO_2_ over CQD@MFe_2_O_4_ is an endothermic process. For example, for CQD@CoFe_2_O_4_, the required energy was calculated (759.38 J/mol), and it is significantly lower than that observed for Ni (2770.27 J/mol) and Zn (2242.95 J/mol) samples (Figure 17a). This behavior is consistent with the experimental trend, where the highest adsorption was seen for CQD@CoFe_2_O_4_. The interaction can be further assessed based on bond lengths and angles. For CQD@CoFe_2_O_4_, the O^–^M^2+^ bond measures 1.69 Å, increasing to 1.78 and 1.84 Å for Ni and Zn ferrites, respectively. CO_2_ molecule geometry (linearity) is decreased to 126° for CQD@CoFe_2_O_4_. For CQD@NiFe_2_O_4_ and CQD@ZnFe_2_O_4_; the angles were 167 and 145°, respectively.

## 3 Materials and Methods

### 3.1 Ferrites Nanoparticles (MFe_2_O_4_ NPs)

**Scheme 1:**
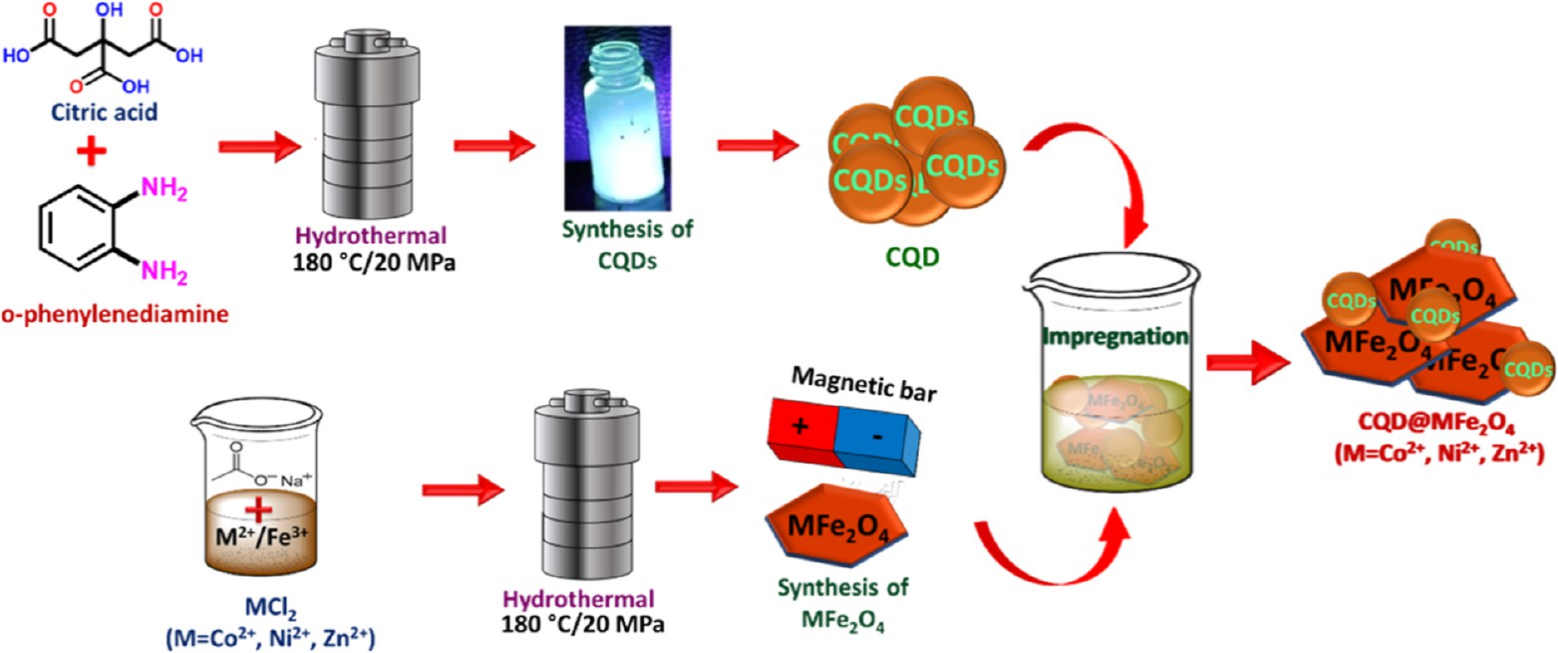
Synthesis of CQD@MFe_2_O_4_

The synthesis of MFe_2_O_4_ NPs (where M = Co^2+^, Ni^2+^, and Zn^2+^) was carried out as detailed elsewhere.^121^ In a typical procedure, metal chloride (0.025 M) and FeCl_3_ (0.05 M) were initially added to a solvent mixture of distilled water and ethylene glycol (1:1, 50 mL). Subsequently, sodium acetate (0.5 M) was introduced to the solution. The resulting mixture was then sonicated for 20 min, transferred to a Teflon-lined stainless-steel autoclave, and heated at 180 °C for 16 h. The dark product obtained was collected using an external magnetic field and washed with distilled water multiple times (Scheme 1).

### 3.2 Carbon Quantum Dots and CQD@MFe_2_O_4_

CQDs were synthesized using a modified hydrothermal method.^157^ Typically, a mixture of citric acid (66.0 mM) and *o*-phenylenediamine (33.0 mM) is combined and then suspended in 50 mL of distilled water. The resulting mixture is heated to 180 °C in an autoclave. After cooling, the CQD product was isolated through efficient centrifugation (see Scheme 1). CQD@MFe_2_O_4_ NPs were prepared using CQD and MFe_2_O_4_ samples as follows: 0.1 g of MFe_2_O_4_ (M = Co^2+^, Ni^2+^, and Zn^2+^) was dispersed in 100 μL of CQDs, followed by the addition of 10 mL of ethanol and sonication for 30 min. The resulting CQD@MFe_2_O_4_ product was isolated using an external magnetic bar and then dried in an oven at 50 °C (see Scheme 1).

### 3.3 Carbon Paste Electrodes

A paste was created by mixing 90 mg of graphite with 10 mg of MFe_2_O_4_ or CD@MF_2_O_4_ in a ratio of 9:1, using 42 mg of paraffin oil. This paste was then packed into a tube measuring 3.0 mm in diameter and 3.0 mm deep to serve as a WE. The modified electrodes used were CPE/CoFe_2_O_4_, CPE/NiFe_2_O_4_, CPE/ZnFe_2_O_4_, CPE/CQD@CoFe_2_O_4_, CPE/CQD@NiFe_2_O_4_, and CPE/CQD@ZnFe_2_O_4_. To prepare the electrode for electrochemical studies, we gently polished it on a damp satin cloth for a few seconds to achieve a smooth surface.

All ferrite samples underwent electrochemical studies, including EIS and polarization, using an ACM Instruments potentiostat (GillAC) with a three-electrode configuration. The acquired impedance spectroscopy and polarization potentiodynamics data were analyzed using EC-Lab V10.40, and the obtained impedance data were extrapolated in the Nyquist model with the Randles model. The EIS frequency range was set from 10 kHz to 0.01 Hz with an AC voltage amplitude of 10 mV. The potentiostat was connected to a three-electrode configuration as follows: (i) the WE was either MFe_2_O_4_ or CD@MF_2_O_4_ paste; (ii) the auxiliary electrode was a platinum filament, and (iii) the reference electrode (RE) was Ag(s)/KCl. The electrolyte used was BRB, and the substrate was [Fe(CN)_6_]^3–^/[Fe(CN)_6_]^4–^ (5.0 mM).

**Scheme 2:**
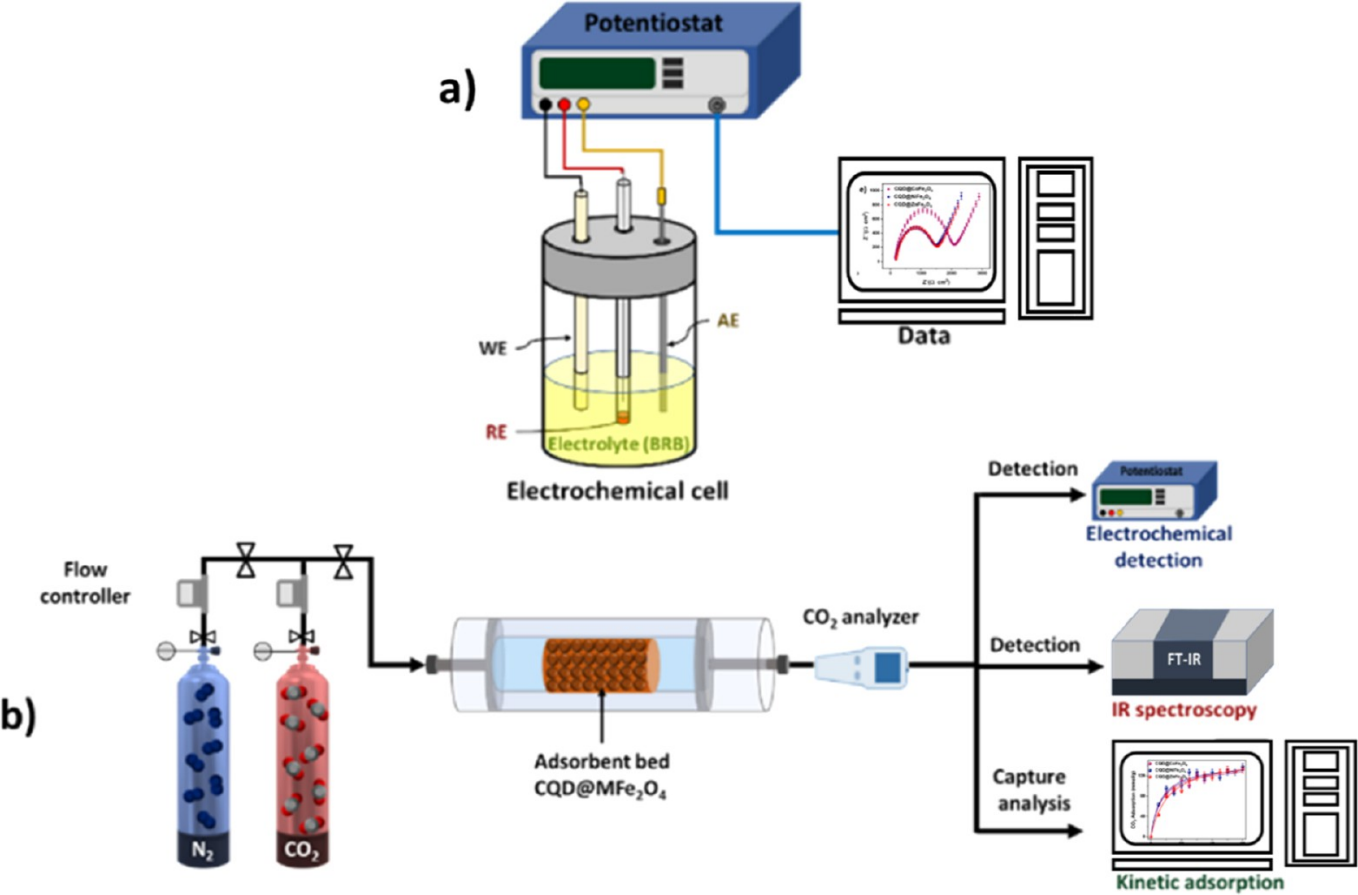
(a) Three-Electrode System Applied for the Electrochemical Analysis and (b) Experimental Setup for the Adsorption of CO_2_

Polarization potentiodynamics data were collected at the circuit potential (*E*) for the RE (SCE) under static conditions at room temperature. The potential range was −0.25 V (SCE) for cathodic and 0.25 V (SCE) for anodic curves, with a scan rate of 10^–3^ V/s. Tafel extrapolation was derived from potentiodynamic polarization curves (polarization currents vs potential). A scheme for the electrochemical analyses and CO_2_ adsorption system is presented in Scheme 2.

### 3.4 Electrochemical CO_2_ Detection

Samples of MFe_2_O_4_ and CQD@MFe_2_O_4_ (where M = Co^2+^, Ni^2+^, and Zn^2+^) underwent a process in which they were placed in a chamber with a CO_2_ flow of 20 mL/min for 1 h to facilitate gas impregnation into the samples. Subsequently, CPE were prepared by blending 90 mg of graphite with 10 mg of CO_2_-exposed MFe_2_O_4_ or CQD@MFe_2_O_4_ in a 9:1 ratio using 42 mg of paraffin oil as a binding agent. The resulting samples were examined using EIS with a GillAC potentiostat from ACM Instruments. The results were then compared to those of samples not exposed to CO_2_.

### 3.5 CO_2_ Capture

The experiments on CO_2_ capture were conducted using samples of MFe_2_O_4_ and CQD@MFe_2_O_4_ (M = Co^2+^, Ni^2+^, and Zn^2+^). The CO_2_ gas was sourced from HAMMOND DRIERITE and flowed at a rate of 20 mL/min into a chamber measuring 16.2 cm × 2.2 cm, where MFe_2_O_4_ and CQD@MFe_2_O_4_ were located. The setup was a homemade system comprising a generator that supplied a continuous stream of CO_2_ gas at a rate of 20 mL/min. The chamber was maintained at a pressure of 1.0 atm and a temperature of approximately 25 °C, with a gas retention time of 15 min. The CO_2_ capture was electrochemically analyzed by using EIS and FTIR methods.

The experiments on CO_2_ capture were conducted using samples of MFe_2_O_4_ and CQD@MFe_2_O_4_ (M = Co^2+^, Ni^2+^, and Zn^2+^). Industrial grade CO_2_ gas (purity of 99%) was obtained from a local company (INFRA, Mexico) and used in the experiment with a constant flow rate of 20 mL/min. The samples were placed for the adsorption studies in the flow chamber (16.2 × Ø 2.2 cm HAMMOND DRIERITE tube). Before the experiments, the samples were subjected to a heat treatment at 200 °C for 3 h to desorb residual gases and ensure a clean surface for adsorption. Each sample was then carefully placed in a glass capsule, which was subsequently placed inside the flow chamber, maintained at a pressure of 1.0 atm at a temperature (approximately 25 °C). Adsorption data were collected over a 60 min retention time, measuring the concentration of CO_2_ at every 15 min time interval. Each experiment was repeated three times to ensure the reproducibility and reliability of the results. CO_2_ capture was analyzed electrochemically using EIS and FTIR methods.

### 3.6 EPR Studies

EPR was conducted at room temperature using a Jeol JES-TE300 spectrometer operating in X-Band mode with a modulation frequency of 100 kHz. The sample solution, along with the spin trapping agent (BMPO, 30 mM), was placed in a quartz tube and inserted into a cylindrical cavity equipped with TE011 to observe the generation of free radicals under UV light (180 mW). Prior to the experiment, the equipment was calibrated by using an external magnetic field (Jeol ES-FC5, a 5350B HP microwave). The EPR data were collected and analyzed using ES-IPRITS-TE software to determine parameters such as *g*-factor values.

### 3.7 Characterization

The samples were analyzed using XRD on a Rigaku RU300 with Cu Kα radiation (λ = 0.154 nm) to determine their crystallite size using Scherrer’s formula. Raman spectra were acquired in an Anton Paar Cora 5001 instrument equipped with a 532 nm (50 mW) laser under STP conditions. Additionally, with using thermogravimetric analysis (TGA, PerkinElmer 4000), the thermal decomposition of the samples was examined in the temperature range of 25–900 °C with a heating rate of 10 °C/min in an air atmosphere. The particle size and crystal planes of CQD@MFe_2_O_4_ were observed using a TEM with a JEOL 2010 at an accelerating voltage of 100 kV. The morphology and elemental composition of the samples were studied using a SEM coupled with the EDS component on a JEOL JSM-5600LV. Furthermore, the oxidation state of the metal ion, chemical composition, and bonding nature of the metal ion were determined through XPS. UV–visible spectra absorption was measured using a spectrophotometer (PerkinElmer 25, 200–700 nm).

### 3.8 DFT Studies

Cobalt ferrite systems CoFe_2_O_4_, NiFe_2_O_4_, and ZnFe_2_O_4_ and their CQD-sensitized counterparts (CQD_2_@CoFe_2_O_4_, CQD_2_@NiFe_2_O_4_, and CQD_2_@ZnFe_2_O_4_) were studied based on the DFT using the PBE functional and LANL2DZ basis set in Gaussian 16 software. Given the large data, a Miztli Supercomputer (256 GB memory, 16 processors) at UNAM was utilized. The excitation energies, minimum energy geometry, and configuration were determined by using a DFT energy/optimization calculation. CDs were optimized from phenylenediamine and citric acid condensation to determine its minimum energy configuration, electronic properties, and potential to be utilized as organic sensitized in light harvesting processes (Scheme S1).

## 4 Conclusions

The different metal ferrites (MFe_2_O_4_, M = Co^2+^, Ni^2+^, and Zn^2+^) prepared are deposited with CQD to increase the CO_2_ adsorption properties. The XRD characteristic peaks of graphitic (19.72° and a weak signal at 41.48°) are seen for CQD@MFe_2_O_4_, observing a monophasic cubic spinel for CoFe_2_O_4_, a reverse cubic spinel structure for NiFe_2_O_4_, and a spinel for ZnFe_2_O_4._ XPS establishes the existence of C, O, and Fe atoms along with specific cations (Co^2+^, Ni^2+^, and Zn^2+^). Electrochemical impedance study found a linear relationship between Z″ (ohm) and Z′ (ohm) at low frequencies, while it is significantly changed to the semicircular loop at higher frequencies, indicating the greater charge transfer resistance (*R*_CT_) than that observed in the low frequencies. This is in agreement with the resistance (1853 Ω cm^2^) of CPE/CoFe_2_O_4_ that decreased to 1652 Ω cm^2^ for CPE/NiFe_2_O_4_ and 1672 Ω cm^2^ for CPE/ZnFe_2_O_4_. Nevertheless, the electron transfer has been improved with lower resistance because of the composite nature of the samples (CQDs@MFe_2_O_4_) as a lower resistance (1163 Ω cm^2^) for CQD@MFe_2_O_4_–CO_2_ when compared to 1567 Ω cm^2^ for MFe_2_O_4_ Thus, the interaction between ferrates with CO_2_ has been enhanced by the existence of CQDs in the samples. This is consistent with the adsorption data of CO_2_ with the samples as *k* = 4.9, *qe* = 121.93 for CQD@CoFe_2_O_4_, *k* = 2.9, *qe* = 156.52 for CQD@NiFe_2_O_4_, and *k* = 3.0, *qe* = 141.71 for CQD@ZnFe_2_O_4_, and it follows the Langmuir pseudo-second-order. The adsorption efficiency is decreased by around 1.0% even after 7 to 8 cycles. The oxygen species (O_2_, O^–^, and O^2–^) react with CO_2_ through electrons from CB to form CO_3_^2–^, which is strongly encouraged by the chemisorption. The positively charged holes generated in the VB of MFe_2_O_4_ NPs can react with H_2_O to generate HO^•^ radicals detected by EPR. Lastly, the interaction of CO_2_ with the ferrites has been further studied by DFT, which shows the CO_2_ adsorption on the surface of the ferrites.

## Data Availability

The data supporting this study’s findings are in the Supporting Information section.

## Supporting Information Available

The preparation and characterization details of samples, XPS, diffuse reflectance spectra of the samples, resistivity of samples during the treatment of CO_2_ at different temperatures, EIS, and DFT studies over the binding of CO_2_ with MFe_2_O_4_ and CQD@MFe_2_O_4_ (M = Co^2+^, Ni^2+^, or Zn^2+^) samples (PDF)
