# Supplementary material for: CO2 Adsorption by Carbon Quantum Dots/Metal Ferrites (M = Co2+, Ni2+, and Zn2+): Electrochemical and Theoretical Studies
Source: ACS Omega. 2025 Apr 3;10(14):13977–4000. doi: 10.1021/acsomega.4c10723 (PMC12004163; doi:10.1021/acsomega.4c10723)
Supplement: Supplementary file 1 — ao4c10723_si_001.pdf [file ao4c10723_si_001.pdf]

CO_2 adsorption by carbon quantum dots/MFe_2O_4 ($M=Co^{2+}$, Ni^{2+} and Zn^{2+}): Electrochemical and theoretical studies

Alan-Javier Santiago-Cuevas[a], Cristian-Brayan Palacios-Cabrera[a], Eduardo Daniel Tecuapa- Flores[b], Ivan J. Bazany-Rodríguez[a], Jayanthi Narayanan[b], Itzia Irene Padilla-Martínez[c], Carlos Alberto Aguilar[d] and Pandiyan Thangarasu[a]*

[a] Universidad Nacional Autónoma de México (UNAM), Facultad de Química, Ciudad Universitaria, 04510 Mexico City, México.

[b] División de Ingeniería en Nanotecnología, Universidad Politécnica del Valle de México, Av. Mexiquense s/n esquina Av. Universidad Politécnica, Tultitlán, Estado de México CP 54910, México

[c] Instituto Politécnico Nacional Unidad Profesional Interdisciplinaria de Biotecnología (UPIBI-IPN), Ciudad de México, Mexico City, México C.P 07738.

[d] Tecnológico de Monterrey, School of Engineering and Sciences, Atlixcáyotl 5718, Puebla, CP 72453, México.

*Email:pandiyan@unam.mx

Supporting information

Table S1. Diffraction pattern peak intensity of the samples.

Peak	Diffraction peak 2θ [°]					
	$ZnFe_2O_4$	Δ2θ	$CoFe_2O_4$	Δ2θ	$NiFe_2O_4$	Δ2θ
(220)	29.93	0.045	30.244	0.074	30.284	0.114
(331)	35.24	0.022	35.473	0.917	35.564	1.056
(222)	36.85	0.049	37.324	1.636	37.205	1.755
(400)	42.85	0.035	43.316	0.316	43.296	0.296

(422)	53.17	0.095	53.896	0.496	53.589	0.189
(511)	56.67	0.083	57.357	0.217	57.317	0.177
(440)	62.19	0.014	63.033	0.243	62.776	0.014
(533)	73.64	0.255	74.701	0.601	74.444	0.344

Table S2. Structural parameters of the samples.

Samples	Lattice constants (a=b=c)	Cell Volume	Density (g/cm^3)	Microstrain (ε) $x10^{-3}$	Dislocation Density (δ) $x10^{-3}$
$CoFe_2O_4$	8.339	579.914	5.376	0.0039	0.0011
$NiFe_2O_4$	8.365	585.298	5.322	0.0073	0.0077
$ZnFe_2O_4$	8.443	601.770	5.324	0.0058	0.0016
CQD@$CoFe_2O_4$	8.441	601.404	5.547	0.0044	0.0018
CQD@$NiFe_2O_4$	8.338	579.656	5.648	0.0086	0.0077
CQD@$ZnFe_2O_4$	8.391	590.887	5.592	0.0061	0.0016

Table S3. Elemental compositions of the samples

Samples	Elemental weigh (%)					
	C	O	Fe	Co	Ni	Zn
CQD	69.88	30.12	-	-	-	-
$CoFe_2O_4$	-	36.42		18.89	-	-
$NiFe_2O_4$	-	31.19	45.40	-	23.42	-
$ZnFe_2O_4$	-	24.45	48.16	-	-	27.40
CQD@$CoFe_2O_4$	19.70	18.23	44.06	18.01	-	-
CQD@$NiFe_2O_4$	8.90	24.40	43.70	-	22.90	-
CQD@$ZnFe_2O_4$	17.90	16.20	44.30	-	-	21.20

Table S4. EDS for MFe_2O_4 and CQD@MFe_2O_4.

Sample	Elemental composition						Ref.
	C	O	Fe	Co	Ni	Zn	
	Wt. %						
$CoFe_2O_4$	-	36.42	44.69	18.89			This work
$NiFe_2O_4$	-	31.19	45.40	-	24.42	-	
$ZnFe_2O_4$	-	24.45	48.16	-	-	27.40	
CQD@$CoFe_2O_4$	19.70	18.23	44.06	18.01	-	-	
CQD@$NiFe_2O_4$	8.90	24.40	43.70	-	22.90	-	
CQD@$ZnFe_2O_4$	17.90	16.20	27.40	-	-	21.20	
$CoFe_2O_4$/AgCl	-	26.49	47.00	26.21	-	-	[1]
$CoFe_2O_4$	-	29.9	44.6	25.5	-	-	[2]
$NiFe_2O_4$	-	46.31	36.24	-	17.45	-	[3]
$NiFe_2O_4$-GO	16.40	34.37	28.80	-	20.44	-	[4]
$ZnFe_2O_4$	-	15.14	55.70	-	-	28.90	[5]
$ZnFe_2O_4$/g-C_3N_4	10.65	20.45	11.34	-	-	57.56	[6]

Table S5. Electrochemical impedance data of CPEs /MFe_2O_4 and CPE/CQD@MFe_2O_4 (M=Co^{2+}, Ni^{2+} and Zn^{2+}).

System	R_s (Ωcm^2)	C_{PE} (Fcm^2) ($x10^{-6}$)	η	R_{CT} (Ωcm^2)	τ (s)	W_0
$CoFe_2O_4$	2073.96575	1.832	0.8347	26214.5875	0.00339	720.10
NiFe2O4	1928.25055	1.500	0.7161	23371.0192	0.00247	913.90
$ZnFe_2O_4$	2148.94541	3.145	0.8494	23653.9613	0.00525	1504.00
CQD@$CoFe_2O_4$	2280.5135	4.438	0.7956	20371.8327	0.00639	652.50
CQ@$NiFe_2O_4$	2375.29911	4.306	0.8093	17132.1454	0.00521	706.20
CQD@$ZnFe_2O_4$	2533.74669	4.085	0.8094	18065.8544	0.00521	611.00

Table S6. Energy and geometrical properties of studied Co, Ni and Zn ferrites and its CQD_2 modified counterparts.

Parameter	Unit	System						
		$CoFe_2O_4$	$NiFe_2O_4$	$ZnFe_2O_4$	CQD_2	CQD_2@ $CoFe_2O_4$	CQD_2@ $NiFe_2O_4$	CQD_2@ $ZnFe_2O_4$
Energy	J/mol	-56163.03	-55684.24	-49446.65	-8902.82	-65221.06	-63591.21	-57713.59
Dipole moment	Dy	5546.05	2510.87	2288.01	7.79	5243.28	1722.93	659.66

Formula		$Co_{32}Fe_{64}O_{128}$	$Ni_{32}Fe_{64}O_{128}$	$Zn_{32}Fe_{64}O_{128}$	$C_{36}H_{48}N_4O_{24}$	$C_{36}H_{48}N_4O_{24}$-$Co_{32}Fe_{64}O_{128}$	$C_{36}H_{48}N_4O_{24}$-$Ni_{32}Fe_{64}O_{128}$	$C_{36}H_{48}N_4O_{24}$-$Zn_{32}Fe_{64}O_{128}$
Reduced Formula		$CoFe_2O_4$	$NiFe_2O_4$	$ZnFe_2O_4$	-	-	-	-
abc	Å	10.56x8.28x 16.56	10.56x8.28x 16.56	10.58x8.29x 16.57	-	-	-	-

Table S7. Bandgap values of bare Co, Ni and Zn ferrites and CDs2 modified Co, Ni and Zn ferrites

Material	Experimental Tauc	Bibliography	DFT HSE1PBE
	E_g (eV)		
CDs2	1.57	~2.40–1.90	Not a semiconductor
$CoFe_2O_4$	1.78	1.17–1.34	1.45
$NiFe_2O_4$	1.83	2.19	2.40
$ZnFe_2O_4$	2.03	~1.90	2.00
$CQD_2@CoFe_2O_4$	1.56	-	1.70
$CQD_2@NiFe_2O_4$	1.70	-	1.70
$CQD_2@ZnFe_2O_4$	1.96	-	1.30

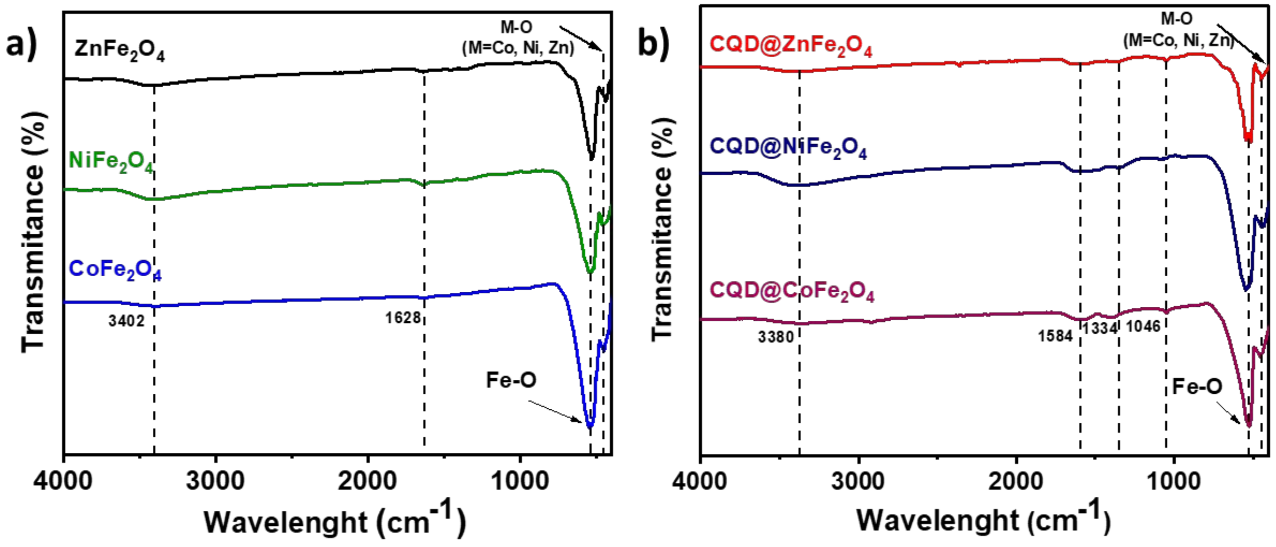

Figure S1. FTIR of a) MFe_2O_4 and b) $CQD@MFe_2O_4$ ($M=Co^{2+}$, Ni^{2+} and Zn^{2+}).

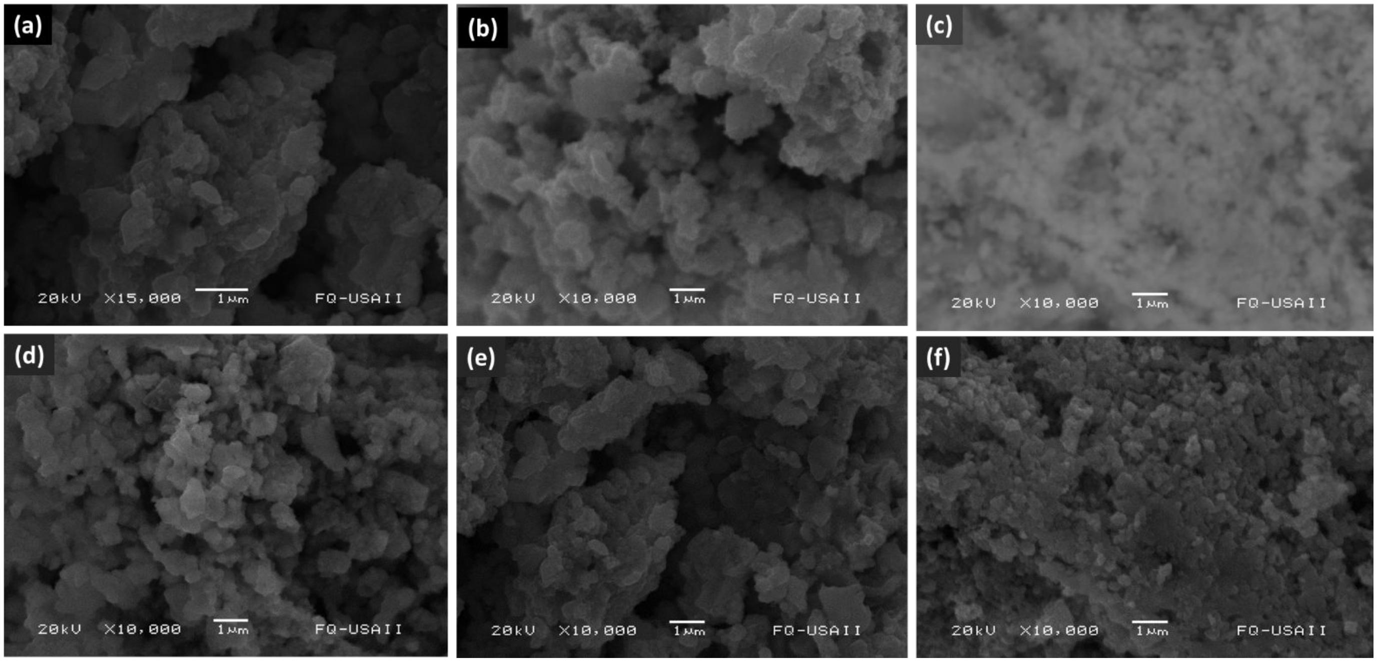

Figure S2. Scanning Electron Microscope of the samples: a) $CoFe_2O_4$, b) CQD@$CoFe_2O_4$, c) $NiFe_2O_4$, d) CQD@$NiFe_2O_4$, e) $ZnFe_2O_4$, f) CQD@ $ZnFe_2O_4$.

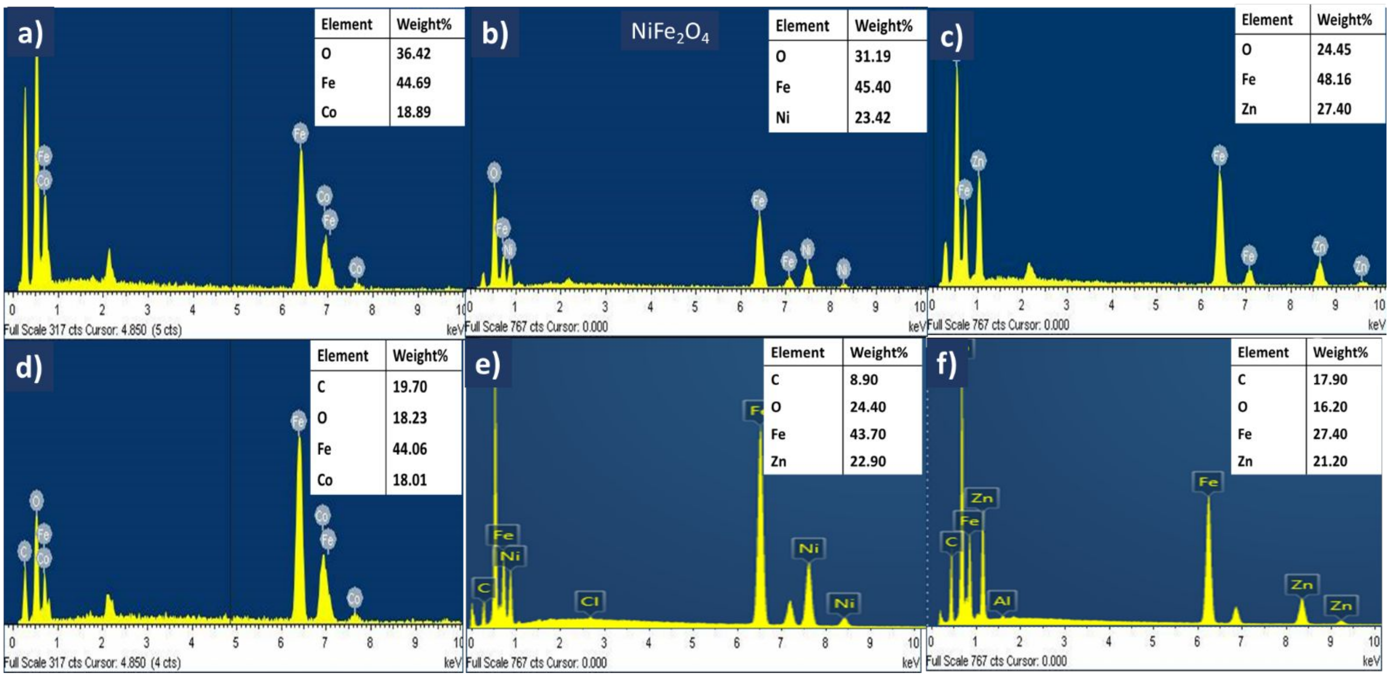

Figure S3. EDS of a) $CoFe_2O_4$; b) CQD@$CoFe_2O_4$, c) $NiFe_2O_4$; d) CQD@$NiFe_2O_4$, a) $ZnFe_2O_4$; b) CQD@$ZnFe_2O_4$.

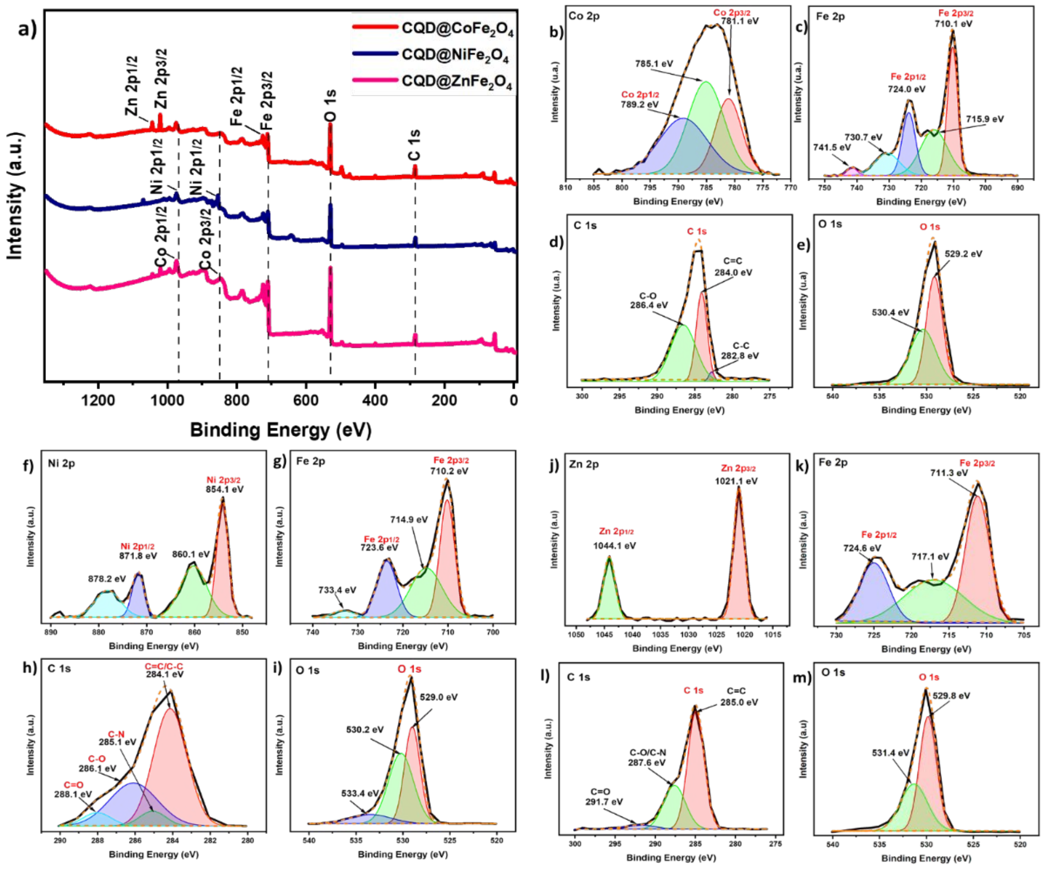

Fig. S4. XPS spectra: a) CQD@$CoFe_2O_4$, b) CQD@$NiFe_2O_4$ y c) CQD@$ZnFe_2O_4$.

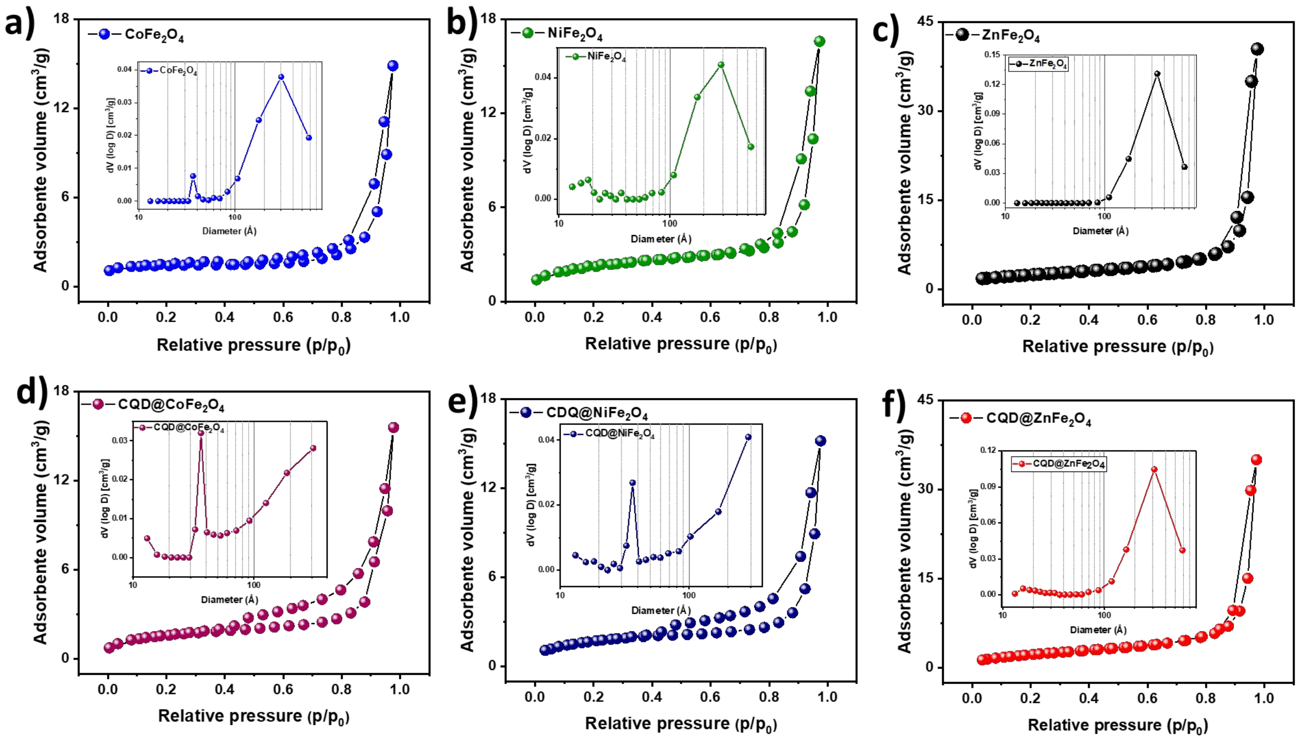

Figure S5. BET Isotherm of a) $CoFe_2O_4$, b) $NiFe_2O_4$, c) $ZnFe_2O_4$, d) CQD@$CoFe_2O_4$, e) CQD@$NiFe_2O_4$ and f) CQD@$ZnFe_2O_4$.

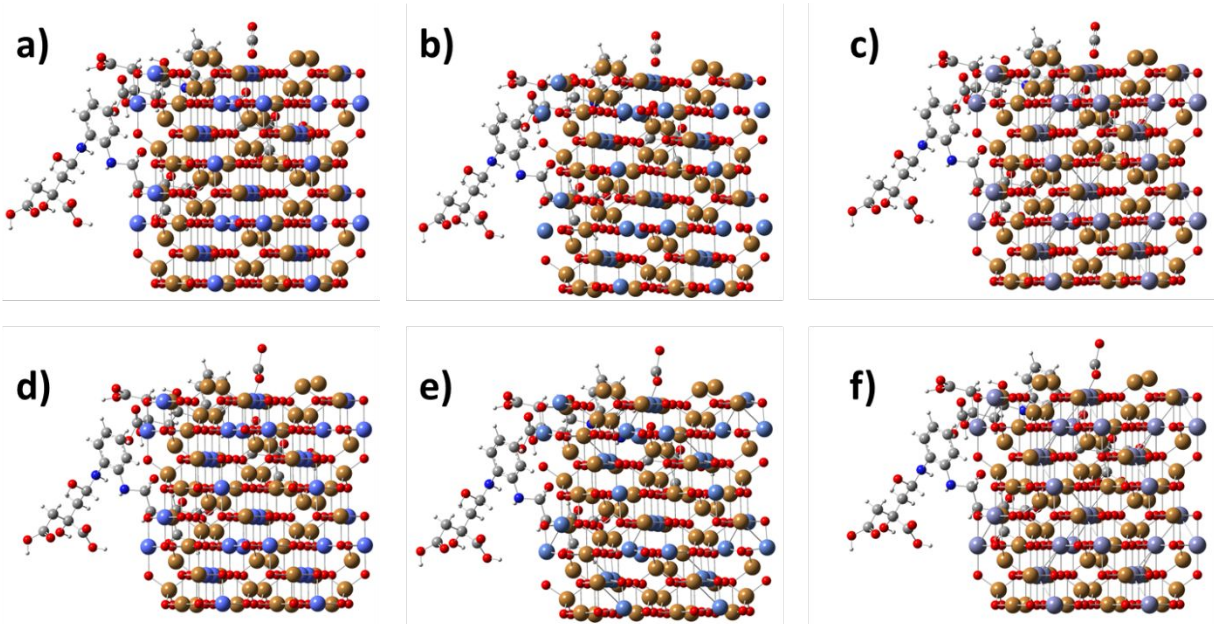

Figure S6. DFT optimization geometries of CDs modified ferrites catalysts in presence of unbound CO_2: a) $CQD_2@CoFe_2O_4$ CO_2; b) $CQD_2@NiFe_2O_4$ CO_2; c) $CQD_2@ZnFe_2O_4$ CO_2 and after adsorption of CO_2: d) $CQD_2@CoFe_2O_4$-CO_2; e) $CQD_2@NiFe_2O_4$-CO_2; f) $CQD_2@ZnFe_2O_4$-CO_2.

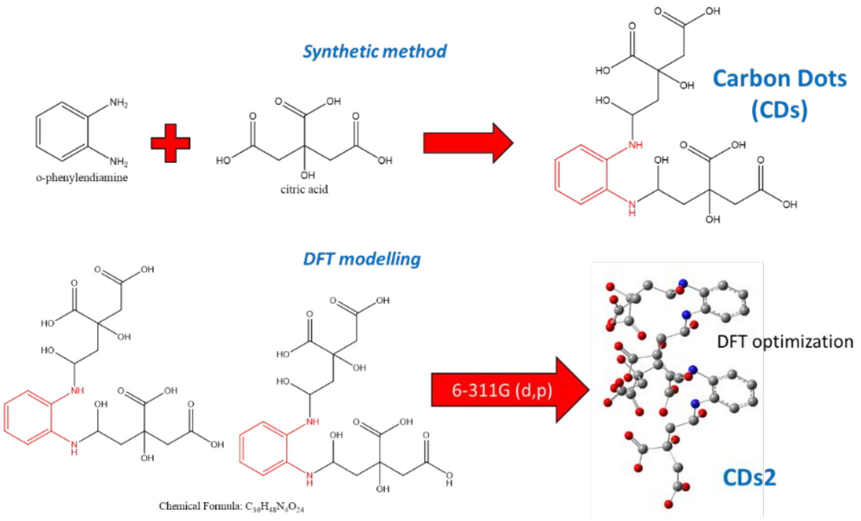

Scheme S1. DFT modeling of carbon quantum dots (CDs)

REFERENCES

1. Brahimi, B.; Mekatel, E.; Mellal, M.; Baaloudj, O.; Brahimi, R.; Hemmi, A.; Trari, M.; Belmedani, M., Enhanced photodegradation of acid orange 61 by the novel hetero-junction $CoFe_2O_4/AgCl$. *Opt. Mater.* **2021,** *121*, 111576 .

2. Al Maashani, M. S.; Khalaf, K. A.; Gismelseed, A. M.; Al-Omari, I. A., The structural and magnetic properties of the nano-$CoFe_2O_4$ ferrite prepared by sol-gel auto-combustion technique. *J Alloy. Compd.* **2020,** *817*, 152786.

3. Morelos-Santos, O.; de la Torre, A. I. R.; Schacht-Hernández, P.; Portales-Martínez, B.; Soto-Escalante, I.; Mendoza-Martínez, A. M.; Mendoza-Cruz, R.; Velázquez-Salazar, J. J.; José-Yacamán, M., $NiFe_2O_4$ nanocatalyst for heavy crude oil upgrading in low hydrogen/feedstock ratio. *Catal Today* **2021,** *360*, 20-26.

4. Aghabeigi, F.; Nikkhah, H.; Zilouei, H.; Bazarganipour, M., Immobilization of lipase on the graphene oxides magnetized with $NiFe_2O_4$ nanoparticles for biodiesel production from microalgae lipids. *Process Biochem.* **2023,** *126*, 171-185.

5. Oliveira, T. P.; Marques, G. N.; Castro, M. A. M.; Costa, R. C. V.; Rangel, J. H. G.; Rodrigues, S. F.; dos Santos, C. C.; Oliveira, M. M., Synthesis and photocatalytic investigation of $ZnFe_2O_4$ in the degradation of organic dyes under visible light. JMR*&T* **2020,** *9* (6), 15001-15015.

6. Renukadevi, S.; Jeyakumari, A. P., Rational design of $ZnFe_2O_4$/g-C_3N_4 heterostructures composites for high efficient visible-light photocatalysis for degradation of aqueous organic pollutants. *Inor.g Chem. Commun.* **2020,** *118, 108047*.
